# Intravenous injection of allogeneic umbilical cord-derived multipotent mesenchymal stromal cells reduces the infarct area and ameliorates cardiac function in a porcine model of acute myocardial infarction

**DOI:** 10.1186/s13287-018-0888-z

**Published:** 2018-05-11

**Authors:** Meikuang Lim, Weiqiang Wang, Lu Liang, Zhi-bo Han, Zongjin Li, Jie Geng, Meng Zhao, Honghong Jia, Jie Feng, Zhe Wei, Baoquan Song, Jiemin Zhang, Jun Li, Tianwen Liu, Fan Wang, Ting Li, Jianming Li, Yihu Fang, Jianhua Gao, Zhongchao Han

**Affiliations:** 1 National Engineering Research Center of Cell Products, Tianjin AmCellGene Engineering Co., Ltd, Tianjin, People’s Republic of China; 2 Beijing Institute of Stem Cells, Health & Biotech Co., Ltd, Beijing, People’s Republic of China; 3 State Key Laboratory of Experimental Hematology, Institute of Hematology and Hospital of Blood diseases, Chinese Academy of Medical Sciences and Peking Union Medical College, Tianjin, People’s Republic of China; 4 Animal Medical Experiment Center, TEDA International Cardiovascular Hospital, Tianjin, People’s Republic of China; 5 Nuclear Medicine Department, TEDA International Cardiovascular Hospital, Tianjin, People’s Republic of China; 6 JiangXi Engineering Research Center for Stem Cell, ShangRao, Jiangxi People’s Republic of China; 7 Institute of Stem Cell, Jiangxi Medical College, ShangRao, Jiangxi People’s Republic of China

**Keywords:** Umbilical cord, Multipotent mesenchymal stromal cell, Porcine, Acute myocardial infarction

## Abstract

**Background:**

Multipotent mesenchymal stromal cell (MSC) therapy has been widely recognized as a feasible strategy for regenerating injured myocardial tissue. However, little is known about the efficacy of intravenous injection of allogeneic umbilical cord (UC) MSCs in preclinical models of porcine myocardial infarction.

**Methods:**

Different dosages of allogeneic UC-MSCs or the vehicle [phosphate-buffered saline (PBS)] were delivered intravenously into an acute myocardial infarction (AMI) porcine model twice after coronary ligation. Echocardiography was performed to examine the cardiac function and single photon emission computed tomography (SPECT) and positron emission tomography (PET)/computed tomography (CT) was performed to detect cardiac perfusion and nonviable myocardium. At the end of the experiment, 2,3,5-triphenyl-tetrazolium chloride (TTC) staining and Masson T staining were performed to determine the infarct area. The protein and gene expression levels associated with cardiac function, inflammation, and angiogenesis were examined by Western blot and real time polymerase chain reaction (PCR). In vivo trafficking of intravenous injection of allogeneic UC-MSCs enhanced green fluorescent protein (eGFP) was detected by real time PCR and immunofluorescence.

**Results:**

After systemic delivery, allogeneic UC-MSCs were largely distributed in the lungs and some in the infracted myocardium. At week 8 following AMI, echocardiography demonstrated significantly improved fractional shortening in the high-dose (1.5 × 10^6^ cells/kg) group. SPECT-PET/CT showed that UC-MSC treatment in both high and low doses markedly ameliorated the left ventricle (LV) infarct area but did not significantly improve the myocardial perfusion defect. LV remodeling was inhibited by UC-MSC therapy, as reflected by a marked reduction in rthe fibrosis area at basal, middle, and apical levels and reduced extracellular matrix deposition in the total myocardial area. Inflammatory biomarkers (tumor necrosis factor alpha and interleukin-6) were reduced and pro-angiogenesis factors (vascular endothelial growth factor and platelet/endothelial cell adhesion molecule 1) were augmented in the myocardial infarct and border area. High-dose UC-MSCs increased the connexin 43 (Cx43) (myocardium preservation) expression in remote area of the LV myocardium after AMI.

**Conclusions:**

Intravenous injection of UC-MSCs is a feasible and effective way to preserve LV function and ameliorate myocardial remodeling in porcine AMI. The cardioprotective effects of UC-MSCs were attributed to paracrine factors that appear to augment angiogenesis, limit inflammation, and preserve Cx43 gap junction.

**Electronic supplementary material:**

The online version of this article (10.1186/s13287-018-0888-z) contains supplementary material, which is available to authorized users.

## Background

Multipotent mesenchymal stromal cells (MSCs) were first described in 1967 by Friedenstein et al., who isolated adherent, fibroblast-like clonogenic cells from bone marrow (BM) called colony-forming unit-fibroblasts (CFU-F). These cells showed a strong capacity for replication in vitro, could differentiate into osteoblasts, chondrocytes, and adipocytes, and supported hematopoietic stroma when a single CFU-F was retransplanted in vivo [1]. However, the heterogeneity of isolation and cultivation procedures among laboratories prompted the International Society for Cellular Therapy (ISCT) to establish criteria for identifying unique populations of MSCs. In 2006, the Tissue Stem Cell Committee of the ISCT proposed minimal criteria to define human MSCs as follows. First, MSCs must be plastic-adherent when maintained under standard culture conditions. Second, MSCs must express CD105, CD73, and CD90, and lack expression of CD45, CD34, CD14 or CD11b, CD79-α or CD19, and HLA-DR surface molecules. Third, MSCs must differentiate to osteoblasts, adipocytes, and chondroblasts in vitro [2]. However, these criteria do not support the purification of homogenous MSC populations. In fact, the isolation of MSCs according to ISCT criteria produces heterogeneous, nonclonal cultures of stromal cells containing stem cells with different multipotential properties, committed progenitors, and differentiated cells. Although the nature and functions of MSCs remain unclear, nonclonal stromal cultures obtained from BM and other tissues that contain a subpopulation of stem cells are currently serving as sources of putative MSCs for therapeutic purposes [3].

MSCs have been successfully isolated from multiple tissues other than BM. The human umbilical cord (UC), whose largest fraction is the Wharton’s jelly, has been characterized as a promising source of MSCs. Compared with any other sources of MSCs, UC-MSCs are easy to obtain from discarded placenta, do not raise any ethical concerns, and the cells embedded are easily isolated without requiring any invasiveness. Moreover, they have a higher MSC yield and shorter MSC doubling time, enabling a faster attainment of the abundant cell numbers required for clinical use while maintaining a safe profile [4–7].

Myocardial infarction (MI) is the most common cause of death globally and is caused primarily by occlusion of the coronary vessels which results in cardiomyocyte death, scar formation, ventricular remodeling, and eventual heart failure. In general, the myocardium does not regenerate lost cardiomyocytes and pharmacological interventions cannot regenerate dead myocardium. Alternatively, rescuing/repairing damaged myocardium by cell therapy such as with MSC transplantation offers the possibility of a new therapeutic option for treating MI and preventing heart failure [8]. Recently, intramyocardial transplantation, intracoronary infusion, and intravenous delivery of allogenic MSCs derived from bone marrow and adipose tissue have been proposed to improve cardiac function in porcine acute myocardial infarction (AMI) [9–12]. A meta-analysis reported that transendocardial injection of BM-MSCs reduced infarct size, whereas direct intramyocardial injection, intravenous infusion, and intracoronary infusion indicated no improvement in porcine models of AMI [13].

A recent study showed that human UC-MSCs (UCX®), obtained using a proprietary technology developed by ECBio, improved the cardiac function after intramyocardial transplantation in a myocardial infarct murine model. The cardioprotective effects of UCX® were attributed to paracrine mechanisms that appear to enhance angiogenesis, limit the extent of the apoptosis, augment proliferation, and activate a pool of resident cardiac Sca-1^+^ progenitor cells (CPCs) [6]. Intracoronary administration of UC-MSCs promoted collateral development and myocardial perfusion, and also reduced fibrosis and apoptosis in a porcine model with chronic myocardial ischemia [14]. In another study, intravenous administration of human UC-MSC exosomes significantly increased left ventricular ejection fraction (LVEF) and left ventricular fractional shortening (LVFS) and reduced cardiac fibrosis in a rat AMI model [15].

Although the beneficial effects of UC-MSCs in the context of a myocardial infarct have been reported, few studies have reported the effect of intravenous administration of UC-MSCs on porcine AMI. The hearts of mini-pigs are similar to those of humans, including heart rate, ratio of heart to body weight, arterial and left ventricular (LV) blood pressure, and LVEF. Compared with other animals, mini-pigs can better reflect the clinical condition in an AMI model [10, 16]. In this study, we utilized a mini-pig AMI model through mid-left anterior descending artery (LAD) ligation to investigate the potential therapeutic effects of low-dose and high-dose intravenous delivery of allogeneic UC-MSCs on AMI. The mechanism underlying the effect of UC-MSC treatment on cardiac function and LV remodeling were also explored.

## Methods

### Isolation and identification of porcine UC-MSCs

Porcine UC was collected after deliveries at the TEDA International Cardiovascular Hospital Animal Medical Experiment Center, Tianjin, China. To isolate UC-MSCs, the UC tissue was dissected into small pieces with sterilized scissors, treated with 1 mg/ml collagenase (Sigma, USA) for 1 h at 37 °C, washed, and then treated with 0.25% trypsin (Invitrogen, USA) for 30 min at 37 °C. After that, the cells were washed and cultured in Dulbecco’s modified Eagle’s medium/nutrient mixture F-12 (DMEM/F12; Invitrogen, USA) supplemented with 10% fetal bovine serum (FBS; Hyclone, Logan, UT, USA), 10 ng/ml basic fibroblast growth factor (bFGF; Peprotech, UK), 10 ng/ml epidermal growth factor (EGF; Peprotech, UK), 100 U/ml penicillin, and 100 μg/ml streptomycin. The medium was changed three times a week, as described previously [17].

The phenotype of porcine UC-MSCs was evaluated by flow cytometric analysis (FACSCalibur, BD, USA) using CD105, CD45, CD34, CD11b, HLA DR (Abcam, USA), CD44 (Biolegend, USA), and CD90 (BD Biosciences, USA) antibodies, coupled to either phycoerythrin (PE) or fluorescein isothiocyanate (FITC).

UC-MSCs were expanded in maintenance medium to passage 6, at which point the cells were used for in vitro adipogenic and osteogenic differentiation assays. Cells were plated at a density of 0.5 × 10^5^ cells in a 24-well plate. After cells became confluent, adipocyte differentiation was induced with the StemPro adipogenesis differentiation kit (Gibco, Grand Island, NY, USA). After 14 days, the cells were fixed with 4% paraformaldehyde (PFA) and stained by Oil Red O (Sigma). Osteogenic differentiation was induced with the StemPro osteogenesis differentiation kit (Gibco, Grand Island, NY, USA) for 28 days. The cells were fixed with 4% PFA and stained with Alizarin Red S (Sigma). All cultures were maintained with 5% CO_2_ in a water jacket incubator at 37 °C.

### Senescence assay of porcine UC-MSCs

Passages 4, 6, and 8 of porcine UC-MSCs were plated in six-well plates at 2.5 × 10^4^ cells/well and incubated at 37 °C with 5% CO_2_ for 72 h, as described previously [18]. The positive control for UC-MSC senescence was established by hydrogen peroxide treatment modified from a previous report [19]. Briefly, passage 6 porcine UC-MSCs were incubated with 4.4 μM H_2_O_2_ for 5 min in complete medium, and then the medium was discarded and the cells were incubated for 48 h in fresh medium before staining. Cells were then stained with a senescence beta-galactosidase staining kit (Beyotime Biotechnology) according to the manufacturer’s recommendation. Senescent cells showed a blue color in the cytoplasm. The percentage of beta-galactosidase-positive cells/500 cells in different microscope fields for each well was determined, as described previously [19, 20].

### Lentivirus-eGFP production and transfection of UC-MSCs

Lentiviruses for gene overexpression of enhanced green fluorescent protein (eGFP) were packaged using the ViralPower Lentivirus Packaging System (Invitrogen) according to the manufacturer’s instruction. To transfect UC-MSCs, lentivirus-eGFP was mixed with DMEM/F12 medium, and the mixture was incubated with UC-MSCs (~ 1 × 10^5^ cells) for 24 h. A multiplicity of transfection of 100 was used. To improve the efficiency of transfection, 3 μg/ml polybrene (Sigma) was added to the transfection medium. Transfection efficiency was detected by flow cytometry and photographed by fluorescence microscope (Olympus IX71). UC-MSCs transfected with lentivirus-eGFP were named UC-MSC-eGFP.

### Animals and study layout

Sixteen female/male Bama mini-pigs with mean weight of 28 kg (range 26–30 kg) underwent surgery. Before surgery, the animals were kept in specific porcine housing facilities at the animal medical experiment center at TEDA International Cardiovascular Hospital. Five pigs were dead because of intractable ventricular fibrillation and could not be resuscitated during or after surgery, and the mortality rate was 31.25%. By design, any animal dying within 24 h of the infarct was removed from the study. Deaths < 24 h after cell delivery were thought to be secondary to the effects of the infarct. Surviving animals were divided into three study groups. The PBS group (*n* = 3) received phosphate-buffered saline (PBS) by intravenous injection, the low-dose group (*n* = 4) received low-dose allogeneic UC-MSCs (0.5 × 10^6^ cells/kg), and the high-dose group (*n* = 4) received high-dose allogeneic UC-MSCs (1.5 × 10^6^ cells/kg). Intravenous injections of allogeneic UC-MSCs were administered twice, 120 min and 4 weeks after AMI induction. Before, during, and after surgery, and at 1 week, 4 weeks, and 8 weeks after AMI induction, these groups underwent transthoracic echocardiography. These groups also underwent Tc-99m sestamibi myocardial perfusion single photon emission computed tomography (SPECT) and ^18^F-fluorodeoxyglucose (FDG) cardiac positron emission tomography (PET)/computed tomography (CT) 1 week, 4 weeks, and 8 weeks after AMI induction. At the end of the experiment, pigs were euthanized with general anesthesia by potassium chloride for removal of the heart. All in vivo experiments were conducted in accordance with the Guide for the Care and Use of Laboratory Animals prepared by TEDA International Cardiovascular Hospital Animal Medical Experiment Center. The study was approved by the Laboratory Animal Welfare Ethics Review Committee of TEDA International Cardiovascular Hospital, Tianjin, China.

### Protocol and procedure of AMI induction and intravenous injection of UC-MSCs

Prior to the surgical operation and imaging examinations, animals were anesthetized with intramuscular (i.m.) administration of atropine 0.5 mg, midazolam 20 mg (Jiangsu Enhua Pharmaceutical Co., Ltd.), and ketamine 100 mg (Fujian Gutian Pharmaceutical Co., Ltd.). An ear vein was cannulated using a 22G venous catheter and anesthesia was maintained with intravenous (i.v.) injection of 5 ml propofol (Xi an Libang Pharmaceutical Co., Ltd.), continued with infusion pumped 14 ml/h propofol as maintenance combined with infusion pumped 50 mg/h lidocaine (China Otsuka Pharmaceutical Co., Ltd.) followed by endotracheal intubation while connecting to a respirator (Servoi, Siemens, USA) and being ventilated mechanically (tidal volume 8–10 ml/kg, frequency 14–18/min). The animals received an intravenous Ringer’s Lactate (China Otsuka Pharmaceutical Co., Ltd.) drip throughout the experiment. Blood pressure and a lead II continuous electrocardiogram (vital signs monitor, Datex-Ohmeda, USA) were recorded.

The heart was exposed through thoracotomy between the fourth and fifth rib under sterile conditions and the mid-LAD was then doubly ligated with 5-0 monofilament polypropylene suture (Prolene, Ethicon, Germany) just beyond the first diagonal branch. A regional myocardial infarct was confirmed by visual inspection for a rapid whitish discoloration of the anterior wall of the left ventricle, followed by a dull discoloration and the development of dyskinetic wall motion. During surgery, AMI was further confirmed by electrocardiography (ECG) with ST elevation appearance, weakness of ventricular wall motion, and decreased ejection fraction which were measured by transthoracic echocardiography under aseptic conditions.

For analgesia, fentanyl 4–8 μg/kg i.v. (Yichang Humanwell Pharmaceutical Co., Ltd.) and succinyl cholinechloride 2.5 mg/kg i.v. (Shanghai Xudong Haipu Pharmaceutical Co., Ltd.) were administered intraoperatively. A single dose of cefuroxime axetil 75 mg (GlaxoSmithKline) was administered prethoracotomy for antibiotic prophylaxis.

To prevent ventricular arrhythmias, amiodarone 6 mg/kg/h i.v. (Cordarone, Sanofi-Winthrop Industrie, France) was administered intraoperatively.

Dexamethasone 5 mg i.v. (Tianjin KingYork Group Co., Ltd.) was administered before intravenous injection of UC-MSCs to prevent an allergic reaction.

After AMI induction, the animals were observed for 120 min to ensure survival. Then, allogeneic UC-MSCs were injected into animals in the treatment groups at the dose of either 0.5 × 10^6^ cells/kg or 1.5 × 10^6^ cells/kg body weight by ear vein. PBS was injected as a negative control.

### Two-dimensional transthoracic echocardiography

Transthoracic echocardiography was performed using a Philips Sonos 5500 ultrasound diagnostic system (USA) equipped with an S4 probe whose frequency is 2.5 to 4.2 MHz. Anesthetized animals were placed in the left lateral decubitus position. Two-dimensional (2D) mode images of parasternal short-axis view were acquired to measure left atrial diameter, and then shifted to position the motion mode (M-mode) cursor at the level of the papillary muscles and perpendicular to the interventricular septum and LV free wall. To evaluate LV structural changes, several parameters from M-mode were measured: left ventricular end-diastolic diameter (LVEDD), left ventricular end-systolic diameter (LVESD), left ventricular diastolic posterior wall thickness (LVPWTd), left ventricular systolic posterior wall thickness (LVPWTs), interventricular septum thickness at diastole (IVSTd), and interventricular septum thickness at systole (IVSTs). Left ventricular fractional shortening (FS) was calculated as an index of systolic function: FS (%) = (LVEDD – LVESD/LVEDD) × 100. Left ventricular end-diastolic volume (LVEDV), left ventricular end-systolic volume (LVESV), and LVEF were determined by modified single-plane Simpson’s rule in the apical four chamber oblique view. Ejection fraction was calculated as an index of systolic function: LVEF (%) = (LVEDV^3^ – LVESV^3^)/LVEDV^3^ × 100. A global diastolic function was assessed using transmitral inflow parameters: peak early (E) and peak late (A) velocities and the E/A ratio [21].

### Tc-99m sestamibi myocardial perfusion SPECT

All pigs underwent technetium (Tc)-99m sestamibi myocardial perfusion SPECT (GE millennium VG-5 dual probe scanner) at the resting state three times, at 1 week, 4 weeks, and 8 weeks after AMI induction. Resting ECG-gated Tc-99m sestamibi SPECT was performed in concordance with the standards of the American Society of Nuclear Cardiology [22]. The pigs were fasted overnight and then Tc-99m sestamibi 370 MBq was injected intravenously at rest. Twenty minutes after the injection, the planar and SPECT images were acquired in the supine position with the ECG-gated technique using eight frames for a cardiac cycle. The SPECT data were acquired using Xeleris workstation Myovation software (GE Medical system, Milwaukee, WI, USA) with low-energy, high-resolution collimator, setting the energy photo-peak at 140 kV with a 20% symmetric window and a 180° acquisition arc. The SPECT acquisition was undertaken in 30 steps and each step collected counts for 25 s. Reconstruction of the images was performed by ordered subsets expectation maximization (OSEM) using a butterworth filter. After reconstruction by OSEM using the butterworth filter, transaxial slices along the vertical long axis, the horizontal long axis, and the short axis were generated.

### ^18^F-FDG cardiac PET/CT

After Tc-99m sestamibi myocardial perfusion SPECT, ^18^F-FDG cardiac PET/CT (FDG-PET) was performed. Imaging was performed on a Discovery PET/CT Elite (Discovery NM 690; GE medical system, Milwaukee, WI, USA). Preparation was performed using simplified glucose/insulin loading (20 g dextrose intravenously with simultaneous and subcutaneous insulin to adjust blood glucose to 5 mmol/l [17, 23]). PET/CT acquisitions for the heart were started at 40 min after the injection of ^18^F-FDG 74–111 MBq/pig. CT images were acquired using parameters with a peak voltage of 140 keV, a tube current of 20 mA × 16 s, a rotation time of 1.0 s, a field of view of 50 cm, and slice thickness of 3.75 mm. Immediately following the CT acquisition, the PET data were acquired in the same anatomic locations in the 2D mode with acquisition times of 10 min. The CT data were used for attenuation correction and the images were reconstructed using a conventional iterative algorithm (OSEM). A Xeleris workstation with Myovation software (GE medical system, Milwaukee, WI, USA) providing multiplanar reformatted images was used for image display and analysis.

### SPECT and PET data analysis

Perfusion SPECT and FDG-PET which were performed on the same day were evaluated simultaneously on the consensus of two independent nuclear medicine physicians. Static tomographic images and polar maps were normalized to their maximum and used for visual analysis of regional perfusion/metabolism patterns using the American Heart Association 17-segment model. The perfusion defect area was quantified on polar maps according to a method described previously [2].Visual classification was performed for normal, matched defect, or mismatch segments. Segments with decreased uptake on perfusion SPECT were visually defined as matched defect segments if FDG uptake was consistent with the decrease on PET. Segments with decreased uptake on perfusion SPECT were defined as mismatched segments if FDG uptake was not consistent with the decrease on PET. SPECT and FDG-PET analysis was managed by Myovation software (GE Medical system, Milwaukee, WI, USA).

### Tissue samples and histology

At the end of experimental week 8, immediately after the imaging examinations, the animals were sacrificed and their hearts were arrested in diastole by i.v. injection of 20 ml 10% potassium chloride (China Otsuka Pharmaceutical Co., Ltd.). The heart was excised and sliced horizontally into 6–7 slices from base to apex. Samples were incubated for 30 min in 1% 2,3,5-triphenyl-tetrazolium chloride (TTC; Sigma-Aldrich, St. Louis, USA) diluted in PBS (pH 7.4) at 37 °C. Stained myocardial samples were photographed from both sides and, in each slide, the infarct area was compared to the total left ventricular area using Image J software (NIH). The calculated infarct area (i.e., the average infarct area) was expressed as the percentage of infarct area compared with the total cross-sectional area of the heart in each section for comparison; i.e., infarct area/(infarct area + noninfarct area) × 100%. The rest of the cardiac tissue was then cut into pieces for specific examinations.

For histopathology, tissues samples were retained from the apex myocardium and fixed in 10% formalin neutral buffer up to 24 h before paraffin embedding and staining with Masson’s trichrome staining. Sections were evaluated under light microscope (Nikon eclipse 80i) and the images were digitized. Six random pictures were taken of each slide at 4× magnification and, for infarct size measurement, the collagen deposition highlighted (blue) was used to define the LV scarred region. The percentage of fibrosis area was calculated by Image J software (NIH).

For other molecular-cellular biological studies, infarct, border (peri-infarct), and remote area from apex region specimens were collected and stored at −80 °C for RNA extraction and Western blot. The peri-infarct area specimens were collected at least 5 mm away from the edge of the infarct area [10].

The technicians/observers who performed the individual image or molecular-cellular biological studies were blinded to the study protocol.

### Quantitative real time PCR assay

Total RNA was isolated from the infarct, border, and a remote area of myocardial tissue samples, and RNA was purified with the E.Z.N.A Total RNA Kit II Omega (Solarbio, Shanghai, China), and cDNA was prepared with the TransScript First-Strand cDNA Synthesis SuperMix (TransGen Biotech, Beijing, China) according to the manufacturer’s recommendation. Quantitative real time polymerase chain reaction (PCR) was performed with the PowerUp SYBR green master mix (Applied Biosystems, Life Technologies, Austin, USA), and the primers (Invitrogen, Shanghai, China) are shown in Additional file 1 (Table S1).

### Western blot analysis of left ventricular specimen

Left ventricular specimens from the apex infarct, border, and remote area were collected, washed, and lysed with RIPA (Solarbio, Beijing, China) supplemented with PMSF (Solarbio, Beijing, China). The total protein was extracted and the protein concentration of the lysates was quantified by the BCA protein assay kit (Solarbio, Beijing, China). Protein (35 μg) was denatured, separated by SDS-PAGE electrophoresis and transferred to a polyvinylidene difluoride (PVDF) membrane (Merck Millipore, USA). The transferred membranes were blocked using 5% bovine serum albumin (BSA) in TBST and incubated with the primary antibodies troponin I (1:200, Santa Cruz), Connexin 43 (Cx43; 1:200, Santa Cruz), tumor necrosis factor (TNF) alpha (1:1000, Abcam), and interleukin (IL)-6 (1:1000, Abcam) overnight and incubated with the corresponding horseradish peroxidase (HRP)-conjugated secondary antibody β-actin (1:1000, Cell Signaling) for 2 h. Bands were visualized using enhanced chemiluminescent (ECL; Merck Millipore, USA) detection reagents and scanned images were quantified using Image J software (NIH). The ratio of target gene to β-actin was used for semiquantification and comparison among different groups.

### In vivo trafficking of intravenous injection of allogeneic UC-MSCs-eGFP

To track UC-MSCs after intravenous transplantation, the AMI pigs were administered with 1.5 × 10^6^ cells/kg UC-MSCs-eGFP 120 min after AMI induction. Cellular engraftment after delivery was assessed by two independent methods: eGFP DNA relative expression level by quantitative real time PCR and immunolabeling of eGFP-positive cells. After 7 days or 14 days, pigs were sacrificed and their multiple organs (heart, spleen, kidney, lung, liver, and skin of the wound surgery area) were rapidly removed and rinsed with PBS, embedded in OCT compound (Sakura Finetek, USA) and snap frozen in liquid nitrogen for immunofluorescence staining. For DNA extraction, tissues from multiple organs were stored at −80 °C.

### DNA extraction

Total DNA was isolated from organ tissue samples (heart, kidney, liver, lung, spleen, and skin of the wound surgery area), and the DNA was purified with the PureLink Genomic DNA mini kit (Invitrogen, Carlsbad, USA) according to the manufacturer’s recommendation. Quantitative real time PCR was performed with the PowerUp SYBR green master mix (Applied Biosystems, Life Technologies, Austin, USA), with the primers (Invitrogen, Shanghai, China) for eGFP being GACGGCCACAAGTTCGTGAT (forward) and GACAAGATGTCCTCGGCGAA (reverse); GAPDH primers were ATTGCCCTCAACGACCACTT (forward) and GGCTCTTACTCCTTGGAGGC (reverse).

### Immunofluorescence staining for eGFP in multiple organs

Fifteen serial sections of multiple organ tissues were prepared at 4 μm thickness by Cryostat (Leica CM1950, Germany) for eGFP immunolabeling, and fixed with 4% PFA, permeabilized in 0.3% Triton, and blocked with 1% BSA. After incubation at 4 °C overnight with rabbit monoclonal anti-GFP antibody (1:400, Molecular Probe, USA), the sections were incubated with goat anti-rabbit IgG (H + L) secondary antibody, Alexa Fluor® 488 conjugate (1:400, Molecular Probe, USA), and counterstained with vectashield mounting medium with DAPI (Vector, USA) and viewed by confocal microscopy (Leica ST2, Germany).

### Statistical analysis

The data are presented as mean ± SD, and comparisons of quantitative data between three groups at different time points were analyzed using general linear model repeated measures. Comparison quantitative data between three or four groups at one time point were analyzed using one-way analysis of variance (ANOVA) with appropriate post-hoc testing. Statistical significance was defined as a *P* value less than 0.05. Statistical analyses were performed using the SPSS software package (SPSS 16.0) for Windows.

## Results

### Intravenous injection of allogeneic UC-MSCs ameliorated cardiac function

**Fig. 1:**
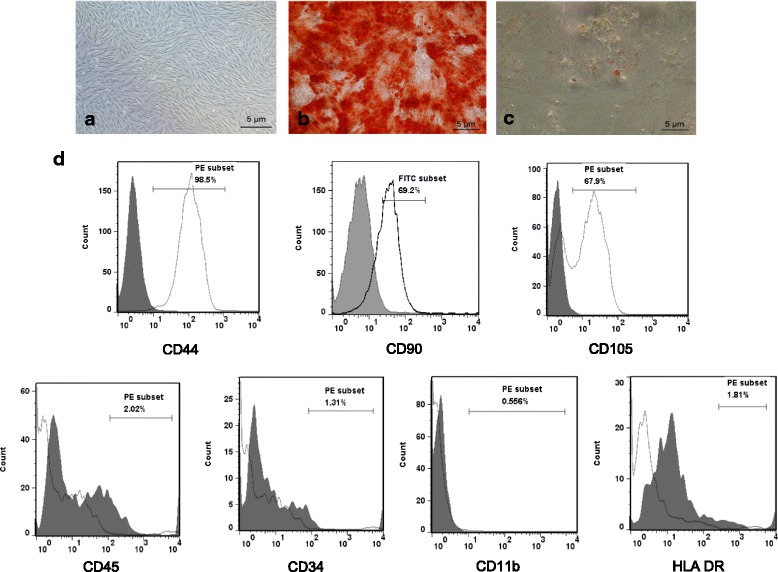
Phenotype of porcine UC-MSCs. **a** Phase-contrast micrographs of porcine UC-MSCs at passage 6. **b** Osteogenic differentiation shown by staining with Alizarin Red. **c** Adipogenic differentiation shown by staining with Oil Red O. Scale bars = 5 μm. **d** Representative results of the flow cytometric analysis of cell surface markers of porcine UC-MSCs at passage 6. White, specific antibodies; grey, isotype control

**Fig. 2:**
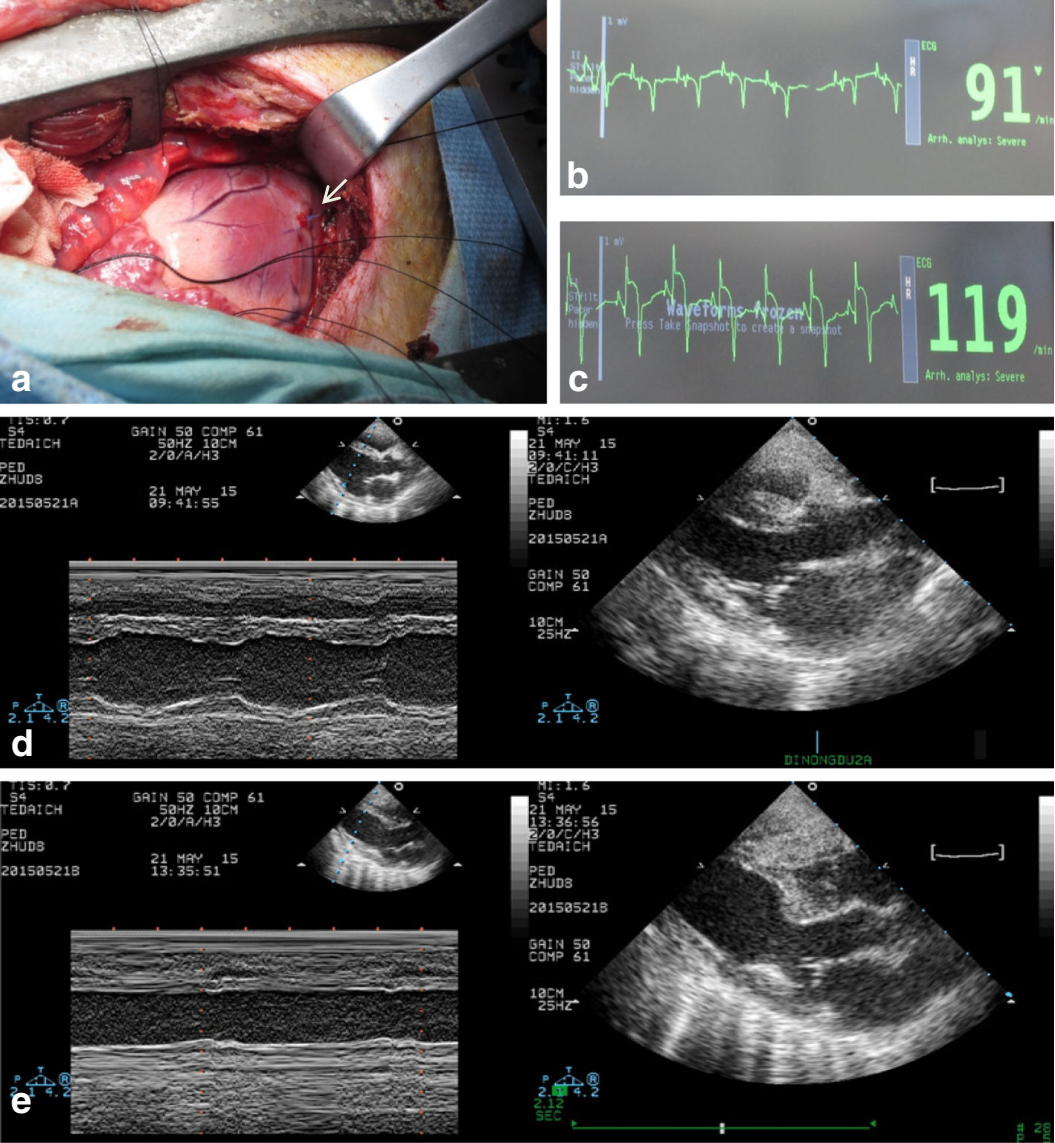
Acute myocardial infarct porcine model based on left anterior descending artery (LAD) ligation. **a** LAD ligation (white arrow). **b** Pre-ligation, showing normal electrocardiography. **c** Post-ligation, showing acute myocardial infarct by ST segment elevation. **d** Pre-ligation M-mode image of 2D parasternal long axis by echocardiography, showing normal echocardiogram. **e** Post-ligation M-mode image of 2D parasternal long axis by echocardiography, showing reduction in left ventricular wall motion

Porcine UC-MSCs were isolated and expanded into passage 6. These cells displayed plastic adherence, fibroblast-like morphology, and differentiated into adipocytes and osteocytes (Fig. 1a–c). The cultured cells expressed high levels of CD90, CD105, and CD44, but not the hematopoietic stem cell and endothelial markers, including CD45 and CD34, and did not express HLA-DR (Fig. 1d). Moreover, passage 6 porcine UC-MSCs showed no significant difference in senescence level compared with passage 4 and 8, which suggests no replicative senescence during in vitro expansion of UC-MSCs. Meanwhile, treatment with hydrogen peroxide significantly increased the senescence level of passage 6 cells (Additional file 2: Figure S1). To evaluate the therapeutic potential of the allogeneic UC-MSCs in an AMI setting, cells were delivered via intravenous injection to pigs subject to permanent ligation of the LAD coronary artery (Fig. 2), 120 min following ligation and 4 weeks later. A regional myocardial infarct was confirmed by visual inspection for a rapid whitish discoloration of the anterior wall of the left ventricle, followed by a dull discoloration and the development of dyskinetic wall motion (Fig. 2a). During surgery, AMI was further confirmed by ECG with ST elevation appearance (Fig. 2b, c) as well as decreased ejection fraction and hypokinetic left ventricular wall motion which were measured by transthoracic echocardiography during aseptic conditions (Fig. 2d, e).

**Fig. 3:**
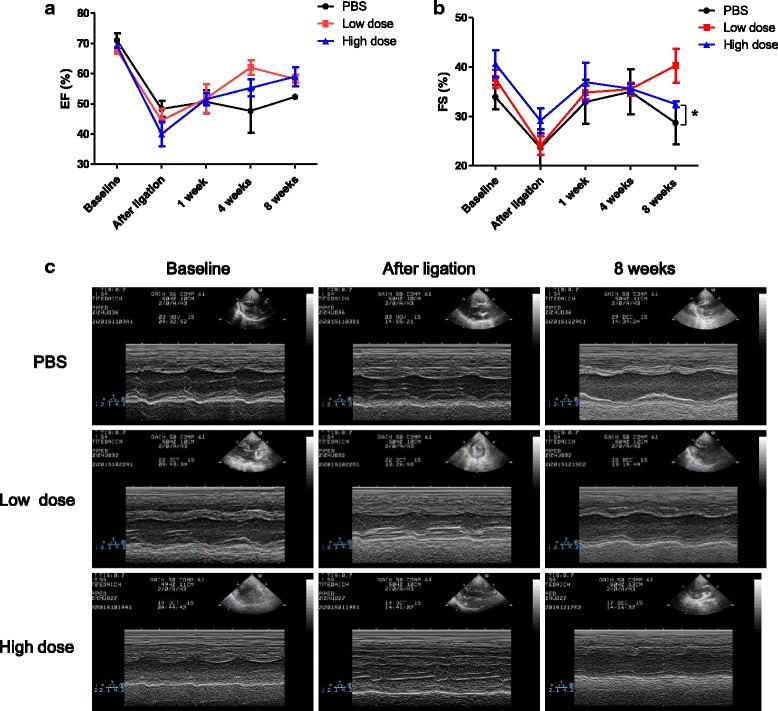
Intravenous injection of allogeneic UC-MSCs improved cardiac function at week 8 after AMI. **a** Left ventricular ejection fraction (EF) and **b** left ventricular fractional shortening (FS) measured by echocardiography. **c** Representative M-mode images of 2D parasternal long axis by echocardiography, showing improvement of left ventricular wall motion in UC-MSC treated group at week 8 after AMI. Data are presented as the mean ± SD. Phosphate-buffered saline (PBS) group, *n* = 3; low-dose group, *n* = 4; and high-dose group, n = 4. *P < 0.05

Infarcted animals that received only the vehicle (PBS) were used as a control. Transthoracic echocardiography demonstrated significantly improved left ventricular FS at week 8 in the high-dose UC-MSC group compared with the PBS group (Fig. 3b). Moreover, although not reaching statistical significance, a tendency to increased LVEF was observed at weeks 4 and 8 in both the low-dose and high-dose UC-MSC groups compared with the PBS group (Fig. 3a). Representative M-mode images of 2D parasternal long axis by echocardiography showed improvement in left ventricular wall motion in the UC-MSC groups at week 8 after AMI (Fig. 3c). In the UC-MSC groups, there was no significant improvement in cardiac structure or left ventricular diastolic function (Additional file 3: Figure S2) compared with the PBS group. These results suggested that intravenous injection of allogeneic UC-MSCs preserved cardiac function after AMI.

### Intravenous injection of allogeneic UC-MSCs did not improve the cardiac perfusion defect, but did reduce left ventricular nonviable myocardium area after AMI

**Fig. 4:**
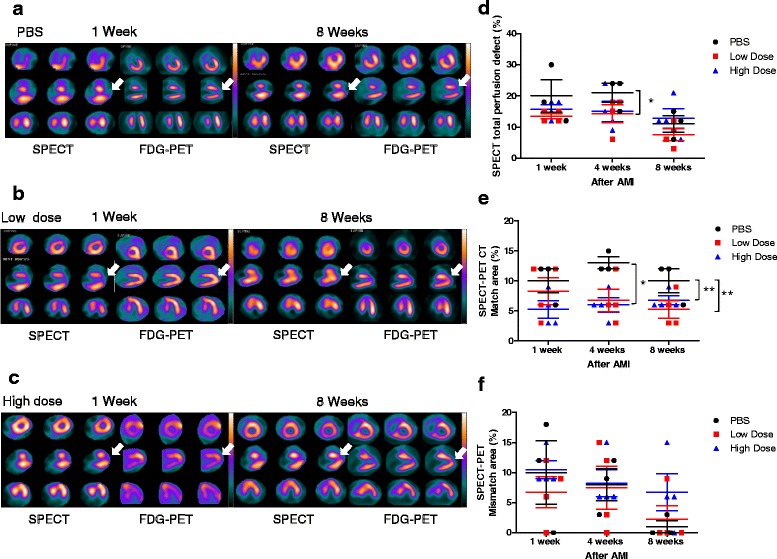
Single photon emission computed tomography (SPECT) and positron emission tomography (PET) analysis demonstrates that intravenous injection of allogeneic UC-MSCs did not improve the myocardial perfusion defect but reduced the left ventricular nonviable myocardium area after acute myocardial infarction (AMI). **a**–**c** Representative SPECT and PET images at weeks 1 and 8. **a** Phosphate-buffered saline (PBS) group: at week 1, SPECT reveals a moderate-severe perfusion defect at the apex and anterior wall apical segment (arrowhead), and PET shows reduced fluorodeoxyglucose (FDG) uptake at the apex and anterior wall apical segment (arrowhead), demonstrating perfusion-metabolism match pattern, which reflects nonviable myocardium; at week 8, SPECT-PET shows the remaining perfusion-metabolism match pattern at the apex and anterior wall apical segment (arrowhead). **b** Low-dose group: at week 1, SPECT shows a moderate-severe perfusion defect at the apex and anterior wall apical segment (arrowhead), and PET demonstrates preserved FDG metabolism at the apex and anterior wall apical segment (arrowhead), showing perfusion-metabolism mismatch pattern, which reflect viable myocardium; at week 8, SPECT-PET shows improvement in myocardial perfusion and FDG metabolism at the apex and anterior wall apical segment (arrowhead). **c** High-dose group: at week 1, SPECT reveals a moderate-severe perfusion defect at the apex and anterior wall apical segment (arrowhead), and PET shows increased FDG uptake at the apex and anterior wall apical segment (arrowhead), demonstrating perfusion-metabolism mismatch pattern, which reflects viable myocardium; at week 8, SPECT shows the remaining perfusion defect at the apex and anterior wall apical segment, and PET shows increased FDG uptake at the apex and anterior wall apical segment (arrowhead), revealing perfusion-metabolism partial mismatch pattern, which expresses partial viable myocardium. **d** Bar chart percentage of SPECT perfusion defect shows that the cardiac perfusion defect was lower in the low-dose group compared with the PBS group at week 4. However, no significant difference was identified between groups at week 8. **e** Percentage of matched segments between SPECT and PET shows lower infarct size in UC-MSC-treated animals groups compared with the PBS group. **f** Percentage of mismatched segments between SPECT and PET showed no significant difference between the three groups. Data are presented as the mean ± SD. PBS group, *n* = 3; low-dose group, *n* = 4; and high-dose group, n = 4. *P < 0.05, **P < 0.01

To evaluate whether the myocardial blood flow perfusion and nonviable myocardium were improved after AMI by two-interval intravenous injections of allogeneic UC-MSCs, we performed SPECT-PET examination at weeks 1, 4, and 8. At the end of this study, representative SPECT and PET demonstrated that the untreated group remained with a moderate-severe myocardial blood flow perfusion defect and nonviable myocardium, reflected by a perfusion-metabolism match pattern in the apex and anterior wall apical segment (Fig. 4a). Treated groups, including the low-dose group, showed amelioration of the myocardial perfusion and increased viable myocardium in the apex and anterior wall apical segment (Fig. 4b). Nevertheless, the high-dose group showed a myocardial perfusion defect and partially viable myocardium (Fig. 4c). Statistical analysis showed that low-dose UC-MSCs reduced the total perfusion defect, and tended to improve myocardial blood flow in the treated groups at week 4. However, no significant difference was observed at weeks 1 and 8 between the three groups (Fig. 4d). The number of match segments between SPECT and PET was significantly reduced in the high-dose UC-MSC group at week 4 and both UC-MSC groups at week 8 compared with the PBS group (Fig. 4e). No significant difference was observed in the number of mismatch segments between SPECT and PET between the groups at weeks 1, 4, and 8 (Fig. 4f). These findings implied that, compared with AMI without treatment, UC-MSC therapy markedly ameliorated the left ventricular infarct area but did not significantly improve the cardiac perfusion defect.

### Intravenous injection of allogeneic UC-MSCs attenuated LV remodeling after AMI

**Fig. 5:**
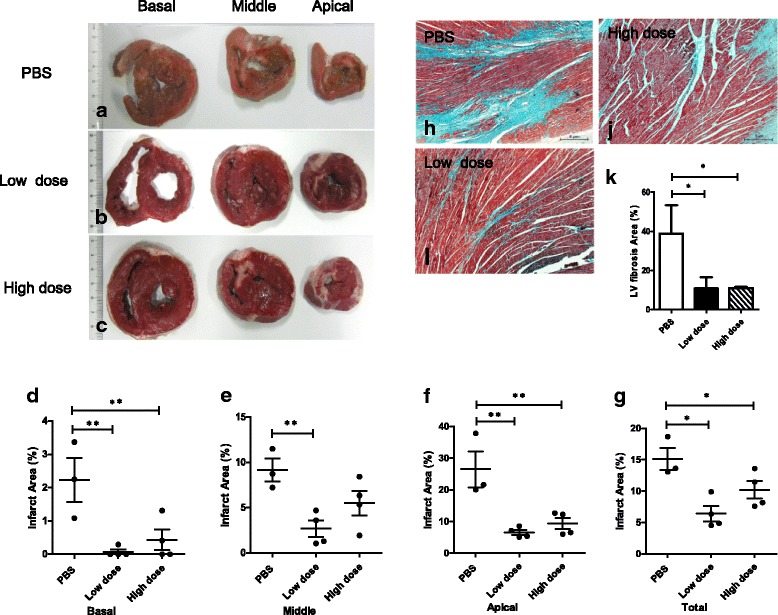
Pathological findings showing that intravenous injection of allogeneic UC-MSCs reduced the infarct area of the left ventricular myocardium at week 8 after AMI. **a**–**c** Representative histological images of left ventricular basal, middle, and apex cross-sections after TTC staining in the three groups. **d**–**g** Infarct area measured by TTC staining was lower in the UC-MSC-treated animals after 8 weeks. **h**–**j** Representative photomicrographs of the left ventricle (LV) highlight the collagen deposition (blue) on the infarct region of the three groups by Masson’s trichrome staining. Scale bars = 5 μm. **k** Quantitative summary of LV infarct area by Masson’s trichrome staining. Data are presented as the mean ± SD. Phosphate-buffered saline (PBS) group, *n* = 3; low-dose group, *n* = 4; and high-dose group, *n* = 4. *P < 0.05, **P < 0.01

TTC staining at week 8 following AMI showed transmural infarct scars, mostly in the apex, anteroseptal, and anterior wall (Fig. 5a–c, Additional file 4: Figure S3). The infarct area was significantly smaller in the UC-MSC groups than the PBS group at the basal, middle, and apical levels (Fig. 5d–g). UC-MSCs significantly reduced the extracellular matrix deposition in the total myocardial area (Fig. 5h–j). Collagen content decreased about 3.5-fold in both low-dose and high-dose UC-MSC groups compared with the PBS group (Fig. 5k). These results indicated that UC-MSCs significantly improved the myocardial remodeling after AMI.

### Intravenous injection of allogeneic UC-MSCs increased Cx43 expression in the remote area of LV myocardium after AMI

**Fig. 6:**
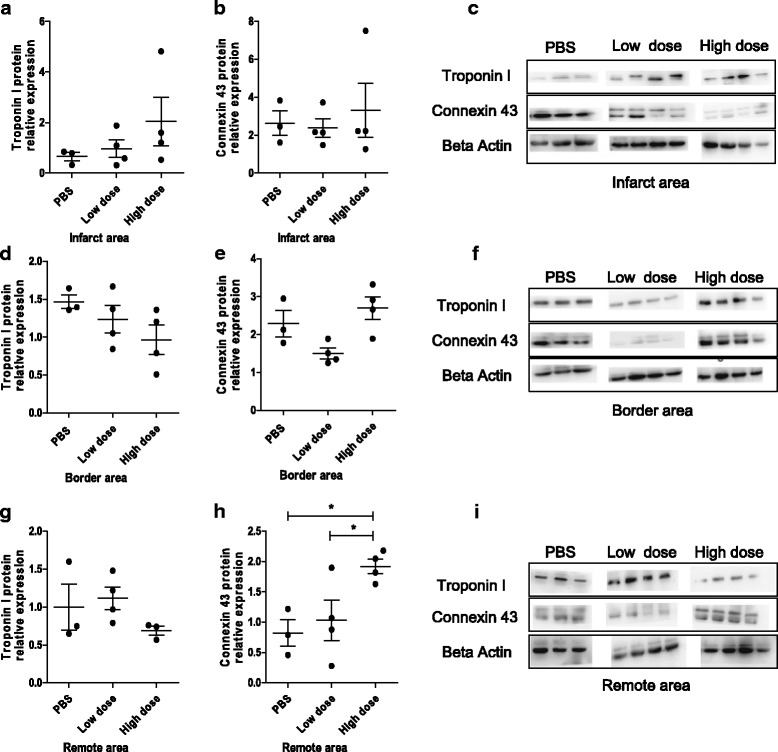
Intravenous injection of allogeneic UC-MSCs increase the protein expression of connexin 43 (Cx43) in the remote area of the LV myocardium at week 8 after AMI. Protein expression of troponin I and Cx43 were measured by Western blot in the infarct area (**a**–**c**), border area (**d**–**f**), and remote area (**g**–**i**). Data are presented as the mean ± SD. Phosphate-buffered saline (PBS) group, *n* = 3; low-dose group, *n* = 4; and high-dose group, *n* = 4. *P < 0.05

The major gap junction protein expressed in the heart, Cx43, demonstrated significantly lower expression in the PBS group and low-dose UC-MSC group than that in the high-dose UC-MSC group at week 8 following AMI induction in the remote area (Fig. 6h, i), but not in the infarct (Fig. 6b, c) or border area (Fig. 6e, f) of the LV myocardium. Cardiac troponins are markers of cardiac muscle damage. There was no significant difference in troponin I protein expression in the three groups in the infarct (Fig. 6a), border (Fig. 6d), or remote areas (Fig. 6g) 8 weeks after AMI. No significant difference in mRNA expression level of troponin I or Cx43 was noted in the infarct, border, or remote area among the three groups (Additional file 5: Figure S4). These results showed that high-dose UC-MSCs prevented downregulation of Cx43 protein expression in the remote area.

### Intravenous injection of allogeneic UC-MSCs inhibited inflammation but promoted angiogenesis in the infarcted myocardium

**Fig. 7:**
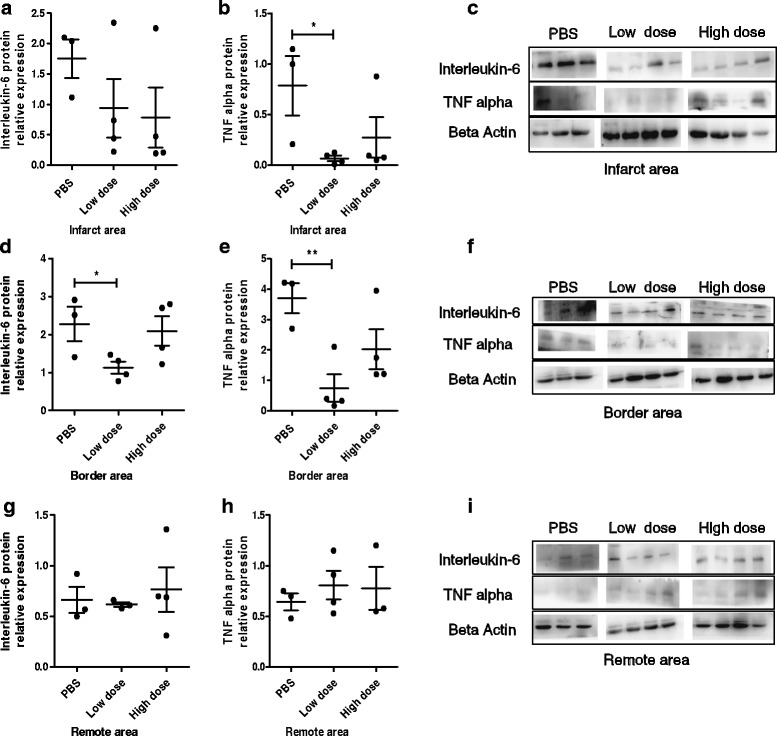
Intravenous injection of allogeneic UC-MSCs decreased inflammatory factors in the infarct area and peri-infarct area of the LV myocardium at week 8 after AMI. Protein expression of tumor necrosis factor (TNF) alpha and interleukin-6 in the infarct area (**a**–**c**), border area (**d**–**f**), and remote area (**g**–**i**). Data are presented as the mean ± SD. Phosphate-buffered saline (PBS) group, *n* = 3; low-dose group, *n* = 4; and high-dose group, *n* = 4. *P < 0.05, **P < 0.01

**Fig. 8:**
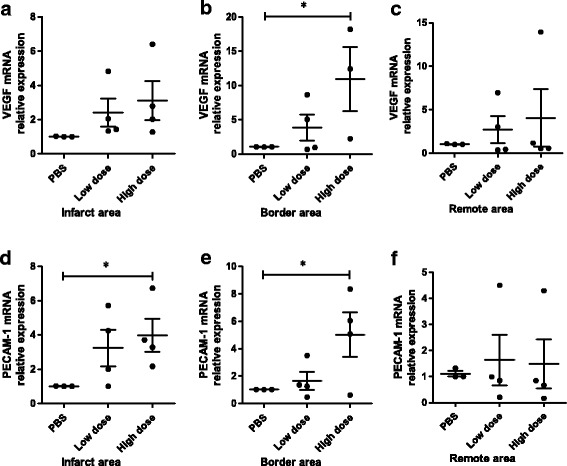
Intravenous injection of allogeneic UC-MSCs increased mRNA expression of angiogenesis biomarkers in the infarct area and peri-infarct area of the LV myocardium at week 8 after AMI. Vascular endothelial growth factor (VEGF) and platelet/endothelial cell adhesion molecule 1 (PECAM-1) mRNA expression in the infarct area (**a**, **d**), border area (**b**, **e**), and remote area (**c**, **f**) of three groups. Data are presented as the mean ± SD. Phosphate-buffered saline (PBS) group, *n* = 3; low-dose group, *n* = 4; and high-dose group, *n* = 4. *P < 0.05

To investigate whether the cardiac improvement observed after AMI could be in part a result of reduced inflammation, improved neovascularization, and increased chemotactic factors, the protein and/or gene expression related to inflammation, angiogenesis, and chemotactic factors were examined in all experimental groups at the end of the study. The protein expression of TNF alpha was significantly lower in the low-dose UC-MSC group than that in the PBS group in the infarct area (Fig. 7b) and border area (Fig. 7e). Additionally, IL-6 protein expression was significantly lower in the low-dose UC-MSC group than that in the PBS group in the border area (Fig. 7d). However, no significant difference in TNF alpha or IL-6 protein expression in the remote area among the three groups was observed (Fig. 7g–i). No significant difference in mRNA expression levels of TNF alpha, IL-6, IL-10, or transforming growth factor (TGF) beta was noted among the three groups in the infarct, border, and remote area (Additional file 6: Figure S5). The mRNA expression level of vascular endothelial growth factor (VEGF) in the border area of the infarct was notably higher in the high-dose UC-MSC group than that in the PBS group (Fig. 8b).The mRNA expression level of platelet/endothelial cell adhesion molecule 1 (PECAM-1; CD31) in both the infarct and border area were significantly higher in the high-dose UC-MSC group than that in the PBS group (Fig. 8d, e). No significant difference in mRNA expression levels of PECAM-1 and VEGF in the remote area of the infarct was observed among the three groups (Fig. 8c, f). The mRNA expression level of the chemotactic factor stromal-derived factor 1-alpha (SDF1-alpha) and its receptor C-X-C chemokine receptor type 4 (CXCR4) in the infarct, border, and remote areas showed no significant difference among the three groups (Additional file 7: Figure S6). These results demonstrated that UC-MSCs promoted generation of the angiogenesis factor and reduced inflammation in the myocardial infarct and border area in a dose-dependent manner.

### In vivo trafficking of intravenous injection of allogeneic UC-MSCs-eGFP in AMI models

Many studies have reported that cells transplanted intravascularly may become entrapped in the lungs, which potentially decreases their therapeutic effect [24]. To investigate the biodistribution of UC-MSCs in AMI, cultured UC-MSCs were transduced with lentivirus-eGFP and transfection efficiency was detected by flow cytometry and fluorescence microscope (Additional file 8: Figure S7). AMI pig models were administered with high-dose UC-MSCs-eGFP 120 min after AMI induction. On day 7, the highest eGFP expression was shown in the lung, followed by the kidney, liver, heart, skin (operation area), and spleen (Additional file 9: Figure S8A). Immunofluorescence detection showed eGFP-positive UC-MSCs in lung tissue (Additional file 9: Figure S8B–D) and peri-infarct heart tissue (Additional file 9: Figure S8E–G). On day 14, we observed that the highest eGFP expression was still in the lung, followed by the spleen, skin (operation area), heart, kidney, and liver (Additional file 10: Figure S9). These results showed that UC-MSCs were well distributed in multiple organs, especially in the lungs, and some homed to the infarcted myocardium.

## Discussion

In the current study, we investigated the therapeutic effect of allogeneic UC-MSCs delivered intravenously at two different times (120 min after LAD ligation and 4 weeks after surgery) in a porcine AMI model. UC-MSCs have been previously shown to preserve cardiac function, differentiate into cardiomyocytes and endothelial cells, and reduce apoptosis and fibrosis after intramyocardial transplantation in AMI murine and porcine models [6, 25], and to improve left ventricular function, perfusion, and remodeling after intracoronary infusion in a porcine model with chronic MI [14]. In this study, we used passage 6 porcine UC-MSCs for AMI treatment. We found that passages 4, 6, and 8 showed relatively low-level senescence and there was no significant difference between the passages. This implies that no obvious replicative senescence occurred during in vitro expansion of UC-MSCs and that passage 6 is usable for therapeutic application in AMI. After two intravenous injections of UC-MSCs, we found that high-dose UC-MSCs improved left ventricular systolic function 8 weeks after AMI. SPECT-PET/CT demonstrated that both low and high dosages of UC-MSCs increased the viable myocardium in the apex and anterior wall apical segment of the infracted heart. Moreover, UC-MSC injections preserved the gap junction integrity in the remote area of the myocardium, reduced inflammation, and increased angiogenesis in infarct and peri-infarct heart.

After a heart attack, stem cell-based therapies during an optimal window of opportunity can exert an optimum therapeutic effect. Some studies reported that intracoronary transplantation of BM-MSCs at 2–4 weeks after MI is more favorable for reduction of the scar area, inhibition of LV remodeling, and recovery of heart function [19]. Intracoronary administration of adipose tissue-derived MSCs 7 days after AMI did not reduce ejection fraction or LV volumes, but effectively increased VEGF expression and neovascularization [9], with better cardiac magnetic resonance-measured perfusion [12]. Intravenously injected MSCs 24 h following MI attenuated the progressive deterioration in LV function and LV remodeling in mice with large infarcts [24]. UC-MSCs from Wharton jelly given intravenously 24–48 h after MI had a significant and durable beneficial effect more than 25 weeks after MI in a rat AMI model [8]. In our study, we administered UC-MSCs 120 min after the AMI model was established but did not find cardiac improvement at week 4 after AMI as measured by echocardiography and SPECT-PET/CT (data not shown). Thus, we designed two intervals of UC-MSC intravenous delivery, at 120 min and at week 4 after AMI. Eight weeks after AMI, we found that the systolic function of FS was improved in the high-dose group. Although there was no statistical significance, the UC-MSC treated groups showed a tendency towards increased EF at week 4 and week 8. A previous study also showed that systemic intravenous delivery of allogeneic BM-MSCs in AMI migrated to the infarcted heart in significant amounts and was effective at increasing fractional shortening, decreasing left ventricular end-diastolic pressure, and therefore improving cardiac function. Moreover, MSCs have the potential to be administered as an allogeneic graft without immunosuppression [9, 12].

Intracoronary application of UC-MSCs in patients with ST elevation AMI increased the myocardial viability as measured by ^18^F-FDG-PET and cardiac perfusion measured by SPECT within the infracted area [2, 3, 7]. In the present study, we demonstrated a reduction in myocardial infarct size and improved cardiac function which was confirmed by the myocardial viability and perfusion examination. We showed that intravenous injection of UC-MSCs significantly increased myocardial viability as measured by ^18^F-FDG-PET at week 8. Although both dosages of UC- MSCs did not increase cardiac perfusion at week 8, a low dose of UC-MSCs increased cardiac perfusion at week 4. A recent study showed that the infarct number of myocardial segments was significantly decreased in the AMI canine model following intracoronary infusion of autologous BM-MSCs, indicating that myocardial regeneration occurred in the infarct area [26].

Intravenous injection of bone marrow-derived MSCs represents the least invasive approach for cellular therapy, and has been previously shown to improve LV function in a closed chest pig model of angioplasty balloon inflation-induced coronary occlusion and reperfusion [27]. In contrast to administration of stem cells by direct intracoronary infusion, some studies reported that the intravenous injection of stem cells relies on entrapment of cells during initial distribution, or on homing and recruitment of cells to the site of infarction, resulted in a lower efficiency of cell uptake [8, 17]. In the present study, we transfected UC-MSCs with lentivirus-eGFP to track the fate of cells after intravenous injection. UC-MSCs were well distributed in many organs, especially in the lungs, with less homing to the infarcted myocardium. Thus, in this porcine model, we speculate that UC-MSC injection preserves cardiac function through paracrine mechanisms involving the reduction of the LV remodeling process and anti-inflammation as well as pro-angiogenesis effects. Paracrine effects seem to be an acceptable explanation because it has been shown that BM-MSCs can secrete a group of cytokines, growth factors, and chemokines, which would beneficially modulate failing myocardium by reducing pathological fibrosis, increasing neovascular formation, reducing cardiomyocyte apoptosis and hypertrophy, attenuating inflammation, and stimulating endogenous stem/progenitor cells for myocardial regeneration [8, 17]. In addition, UC-MSCs (Wharton jelly MSCs) can secrete large amounts of anti-apoptotic, angiogenic factors, and growth factors for the regeneration of myocardium and coronary vessels, augment proliferation, and activate a pool of resident cardiac Sca-1^+^ progenitor cells [6, 27]. In our study, we demonstrated that UC-MSCs attenuated inflammation and increased angiogenesis in the infarct and peri-infarct area of the infarcted heart. Moreover, we also revealed that Cx43 expression was increased in the remote area of the infarcted myocardium following intravenous injection of UC-MSCs. Abnormal expression of Cx43 in the heart has been reported in several forms of cardiomyopathies such as hypertrophic, dilated, and ischemic cardiomyopathy. Commonly, Cx43 is downregulated and heterogeneously redistributed throughout the heart. This abnormal Cx43 expression is often followed by a reduction in the conduction velocity, which then seems to be accompanied by an increase in fibrosis. All these disturbances may lead somehow to the development of arrhythmias [27]. A previous study suggested that improved LV function may be attributable, at least partly, to the upregulation of Cx43 that was identified by Western blot following BM-MSC transplantation [10].

Intravenous injection of allogeneic MSCs had dose-dependent beneficial effects on cardiac function, which was statistically significant at 1 × 10^5^ and 1 × 10^6^ cells per kg bodyweight [9]. We showed that 1.5 × 10^6^ cells per kg bodyweight preserved cardiac function, reduced LV fibrosis, and augmented angiogenesis. Although a low dose of 0.5 × 10^6^ cells per kg bodyweight did not significantly improve myocardial function, reduced myocardial fibrosis and attenuated inflammation in the infarct and peri-infarct area of the MI were noted.

## Conclusions

In this study, we demonstrate that intravenous injection of allogeneic UC-MSCs prevented LV remodeling and improved LV functions in an AMI porcine model. The cardioprotective effects of UC-MSCs might be exerted by enhancing angiogenesis, attenuating inflammation, reducing fibrosis, and upregulating Cx43 gap junction expression, which are mediated by paracrine factors secreted by UC-MSCs.

## Additional files

Additional file 1: **Table S1.** Primers used in this study (DOCX 17 kb)

Additional file 2: **Figure S1.** Analysis of senescence in porcine UC-MSCs. Representative microphotographs of porcine UC-MSCs in passage 4 (A), passage 6 (B), passage 8 (C), and passage 6 with peroxide hydrogen treatment as positive control (D) after β-galactosidase staining. Scale bar = 5 μm. Quantitative summary of senescence porcine UC-MSCs (E). Data are presented as the mean ± SD (*n* = 6 per group) and represent two independent experiments. **P < 0.01, ***P < 0.001. (PPTX 1750 kb) (PPTX 1750 kb)

Additional file 3: **Figure S2.** Effects of intravenous injection of allogeneic UC-MSCs on cardiac structure and diastolic function after AMI. Left ventricular end-diastolic diameter (A), left ventricular end-systolic diameter (B), left ventricular end-diastolic volume (C), and left ventricular end-systolic volume (D). Intraventricular septal diastolic thickness (E), intraventricular septal systolic thickness (F), left ventricular posterior wall diastolic thickness (G), and left ventricular posterior wall systolic thickness (H). Left atrial diameter (I). Cardiac diastolic function: E/A ratio (J). Data are presented as the mean ± SD (PBS group *n* = 3, low-dose group *n* = 4, and high-dose group *n* = 4). *P < 0.05 (ZIP 298 kb)

Additional file 4: **Figure S3.** Histological images of left ventricular basal, middle, and apex cross-sections after TTC staining in the three groups PBS (*n* = 3), low-dose group (*n* = 4), and high-dose group (*n* = 4) at 8 weeks follow-up. Schematic infarct distribution shows that the infarction (white color) is located in the anterior and anteroseptal segments of the heart. (PPTX 6390 kb)

Additional file 5: **Figure S4.** Intravenous injection of allogeneic UC-MSCs did not impact mRNA expression levels of the cardiac function biomarkers troponin I and connexin 43 in the infarct area (A,D), border area (B,E), or remote area (C,F) of LV myocardium at week 8 after AMI. (PPTX 157 kb)

Additional file 6: **Figure S5.** Intravenous injection of allogeneic UC-MSCs did not affect mRNA expression level of the inflammation factors TNF alpha, IL-6, IL-10, and TGF-beta in the infarct area (A,D,G,J), border area (B,E,H,K), or remote area (C,F,I,L) of LV myocardium at week 8 after AMI. (ZIP 248 kb)

Additional file 7: **Figure S6.** Intravenous injection of allogeneic UC-MSCs did not affect mRNA expression of the chemotaxis factors SDF1-alpha and its receptor CXCR4 in the infarct area (A,D), border area (B,E), or remote area (C,F) of LV myocardium at week 8 after AMI. (PPTX 151 kb)

Additional file 8: **Figure S7.** Transfer of lentivirus-eGFP into UC-MSCs. Representative phase-contrast (A) and fluorescence (B) microscopic photograph of UC-MSCs transduced with lentivirus-eGFP. (C,D) Flow cytometric analysis of the transfection efficiency of lentivirus-eGFP into UC-MSCs. Scale bar = 5 μm. Data are representative of three independent experiments. (PPTX 795 kb)

Additional file 9: **Figure S8.** In vivo trafficking of intravenous injection of allogeneic UC-MSCs-eGFP 7 days after AMI induction. (A) Biodistribution pattern of UC-MSCs-eGFP measured by DNA relative expression of eGFP in many tissues (heart, lung, liver, spleen, kidney, wound operation of skin). (B–D) Immunofluorescence on eGFP (green) and nuclei (blue) in lung tissue. (E–G) Immunofluorescence on eGFP (green) and nuclei (blue) in peri-infarct heart tissue. Scale bar = 20 μm. (PPTX 783 kb)

Additional file 10: **Figure S9.** In vivo trafficking of intravenous injection of allogeneic UC-MSCs-eGFP 14 days after AMI induction. (A) Biodistribution pattern of UC-MSCs-eGFP measured by DNA relative expression of eGFP in many tissues (heart, lung, liver, spleen, kidney, wound operation of skin). (B–D) Immunofluorescence on eGFP (green) and nuclei (blue) in lung tissue. (E–G) Immunofluorescence on eGFP (green) and nuclei (blue) in kidney tissue. Scale bar = 20 μm. (PPTX 1467 kb)

## Abbreviations

AMI: Acute myocardial infarction
BM: Bone marrow
BSA: Bovine serum albumin
CFU-U: Colony-forming unit-fibroblasts
CT: Computed tomography
Cx43: Connexin 43
CXCR4: C-X-C chemokine receptor type 4
DMEM: Dulbecco’s modified Eagle’s medium
ECG: Electrocardiography
EF: Ejection fraction
eGFP: Enhanced green fluorescent protein
FDG: Fluorodeoxyglucose
FS: Fractional shortening
IL: Interleukin
ISCT: International Society for Cellular Therapy
IVSTd: Interventricular septum thickness at diastole
IVSTs: Interventricular septum thickness at systole
LAD: Left anterior descending artery
LV: Left ventricular
LVEDD: Left ventricular end-diastolic diameter
LVEDV: Left ventricular end-diastolic volume
LVEF: Left ventricular ejection fraction
LVESD: Left ventricular end-systolic diameter
LVESV: Left ventricular end-systolic volume
LVPWTd: Left ventricular diastolic posterior wall thickness
LVPWTs: Left ventricular systolic posterior wall thickness
MI: Myocardial infarction
MSC: Mesenchymal stromal cell
PBS: Phosphate-buffered saline
PCR: Polymerase chain reaction
PECAM-1: Platelet/endothelial cell adhesion molecule 1
PET: Positron emission tomography
PFA: Paraformaldehyde
SDF1-alpha: Stromal-derived factor 1-alpha
SPECT: Single photon emission computed tomography
TGF: Transforming growth factor
TNF: Tumor necrosis factor
TTC: 2,3,5-Triphenyl-tetrazolium chloride
UC: Umbilical cord
VEGF: Vascular endothelial growth factor
