# Supplementary material for: Intravenous injection of allogeneic umbilical cord-derived multipotent mesenchymal stromal cells reduces the infarct area and ameliorates cardiac function in a porcine model of acute myocardial infarction
Source: Stem Cell Res Ther. 2018 May 11;9:129. doi: 10.1186/s13287-018-0888-z (PMC5948807; doi:10.1186/s13287-018-0888-z)
Supplement: Supplementary file 1 — Table S1. Primers used in this study (DOCX 17 kb) [file 13287_2018_888_MOESM1_ESM.docx]

**Additional file 9: Table S1. Primers used in this study**

| **Gene** | **Forward** | **Reverse** |
| --- | --- | --- |
| *Troponin I* | 5’-GGACACGGAGAAGGTGAGTG-3’ | 5’- CTTCCTGCCGAGGACCCTTA-3’ |
| *Connexin 43* | 5’-GAGGTGGCCTTCTTGCTGAT-3’ | 5’-GTTCAAGGCGAGAGACACCA-3’ |
| *TNF alpha* | 5’-GCCCTTCCACCAACGTTTTC-3’ | 5’-CAAGGGCTCTTGATGGCAGA-3’ |
| *IL-6* | 5’-TGCAGTCACAGAACGAGTGG-3’ | 5’-CAGGTGCCCCAGCTACATTAT-3’ |
| *IL-10* | 5’-TAGGGTGTGCCCTATGGTGT-3’ | 5’-GGGTGGGTAGGCTTGGAATG-3’ |
| *VEGF* | 5’-GACCAGAAACCCCACGAAGT-3’ | 5’-CACACAGGACGGCTTGAAGA-3’ |
| *PECAM-1* | 5’-CGAGGTCTGGGAACAAAGGG-3’ | 5’-CTGCTCTGCGGTCCTAAGTC-3’ |
| *SDF1 alpha* | 5’-CCATGGGTGTCAAGGTCCTC-3’ | 5’-CAGGCTGACCGGTTTCTCAT-3’ |
| *CXCR4* | 5’-GCGCAAAGCTCTCAAAACCA-3’ | 5’-CAGTGGAAAAAGGCAAGGGC-3’ |
| *GAPDH* | 5’-ATTGCCCTCAACGACCACTT-3’ | 5’-GGCTCTTACTCCTTGGAGGC-3’ |
