# Supplementary figures and images for: Intravenous injection of allogeneic umbilical cord-derived multipotent mesenchymal stromal cells reduces the infarct area and ameliorates cardiac function in a porcine model of acute myocardial infarction
Source: Stem Cell Res Ther. 2018 May 11;9:129. doi: 10.1186/s13287-018-0888-z (PMC5948807; doi:10.1186/s13287-018-0888-z)

## Slide 1
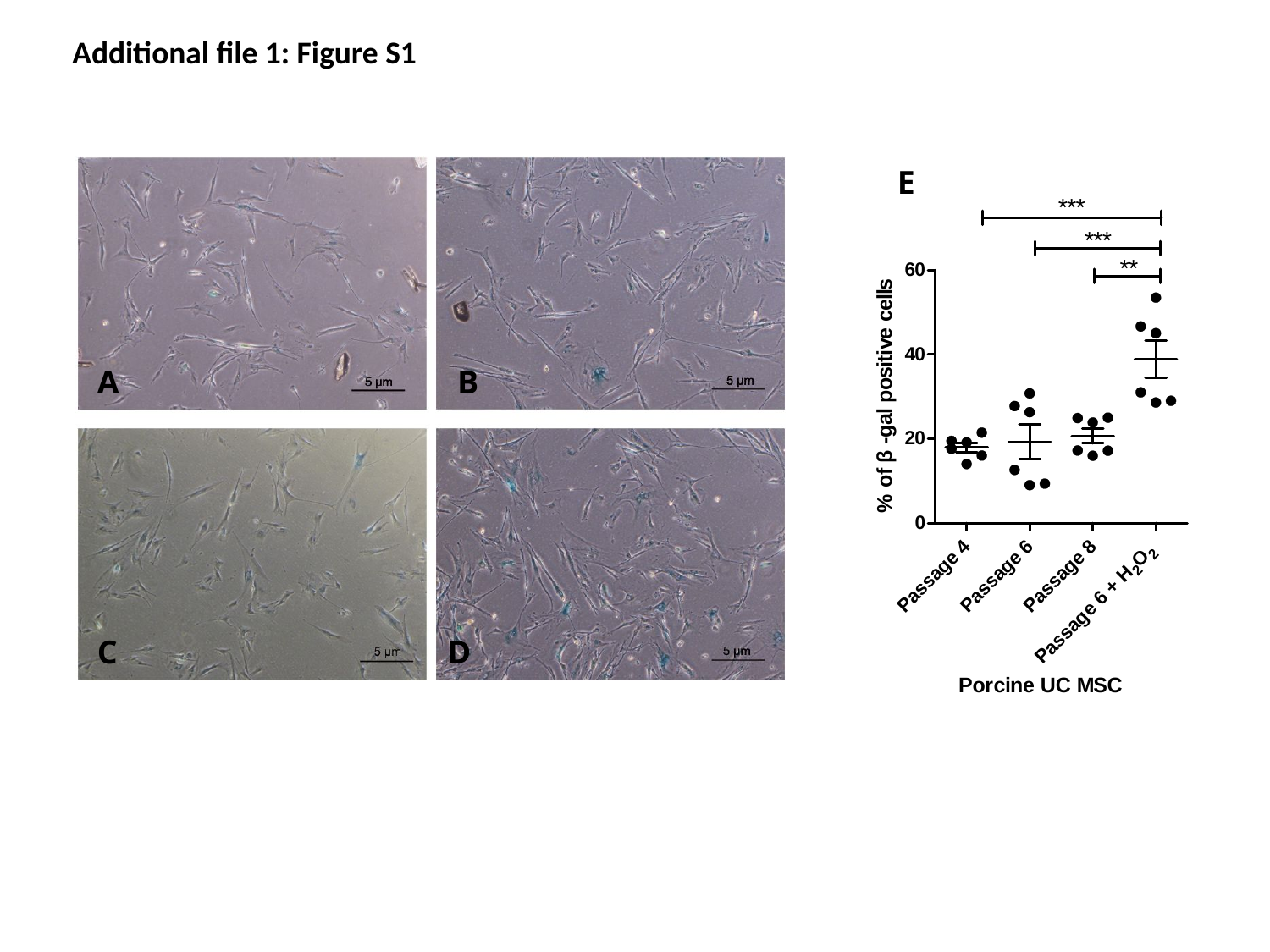

Additional file 1: Figure S1
E
A
B
C
D

Supplement: Supplementary file 2 — Figure S1. Analysis of senescence in porcine UC-MSCs. Representative microphotographs of porcine UC-MSCs in passage 4 (A), passage 6 (B), passage 8 (C), and passage 6 with peroxide hydrogen treatment as positive control (D) after β-galactosidase staining. Scale bar = 5 μm. Quantitative summary of senescence porcine UC-MSCs (E). Data are presented as the mean ± SD (n = 6 per group) and represent two independent experiments. **P < 0.01, ***P < 0.001. (PPTX 1750 kb) (PPTX 1750 kb) [file 13287_2018_888_MOESM2_ESM.pptx]

## Slide 1
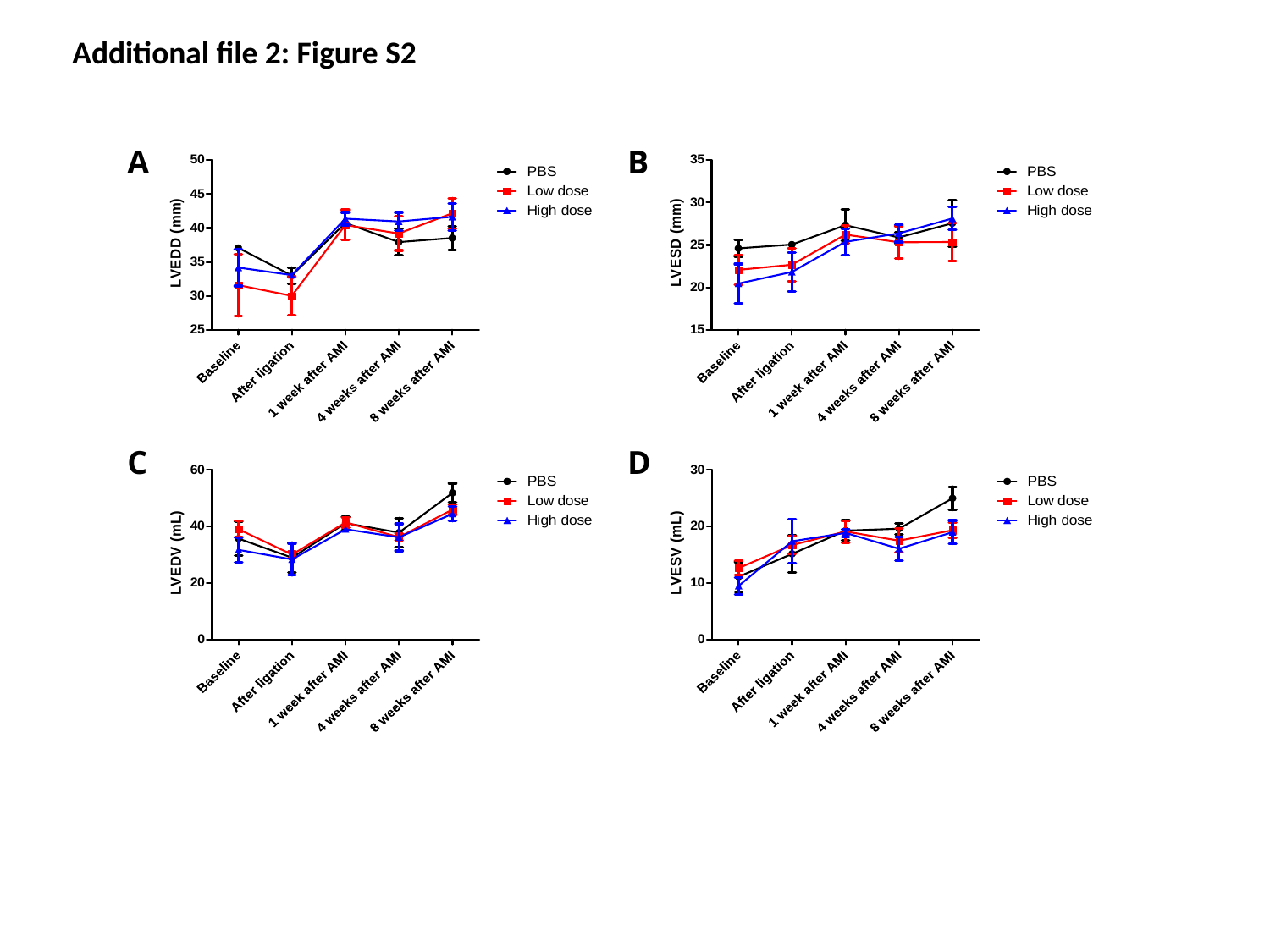

Additional file 2: Figure S2
A
B
C
D

Supplement: Supplementary file 3 — Figure S2. Effects of intravenous injection of allogeneic UC-MSCs on cardiac structure and diastolic function after AMI. Left ventricular end-diastolic diameter (A), left ventricular end-systolic diameter (B), left ventricular end-diastolic volume (C), and left ventricular end-systolic volume (D). Intraventricular septal diastolic thickness (E), intraventricular septal systolic thickness (F), left ventricular posterior wall diastolic thickness (G), and left ventricular posterior wall systolic thickness (H). Left atrial diameter (I). Cardiac diastolic function: E/A ratio (J). Data are presented as the mean ± SD (PBS group n = 3, low-dose group n = 4, and high-dose group n = 4). *P < 0.05 (ZIP 298 kb) [file 13287_2018_888_MOESM3_ESM.zip › Additional file 2 Figure S2.pptx]

## Slide 1
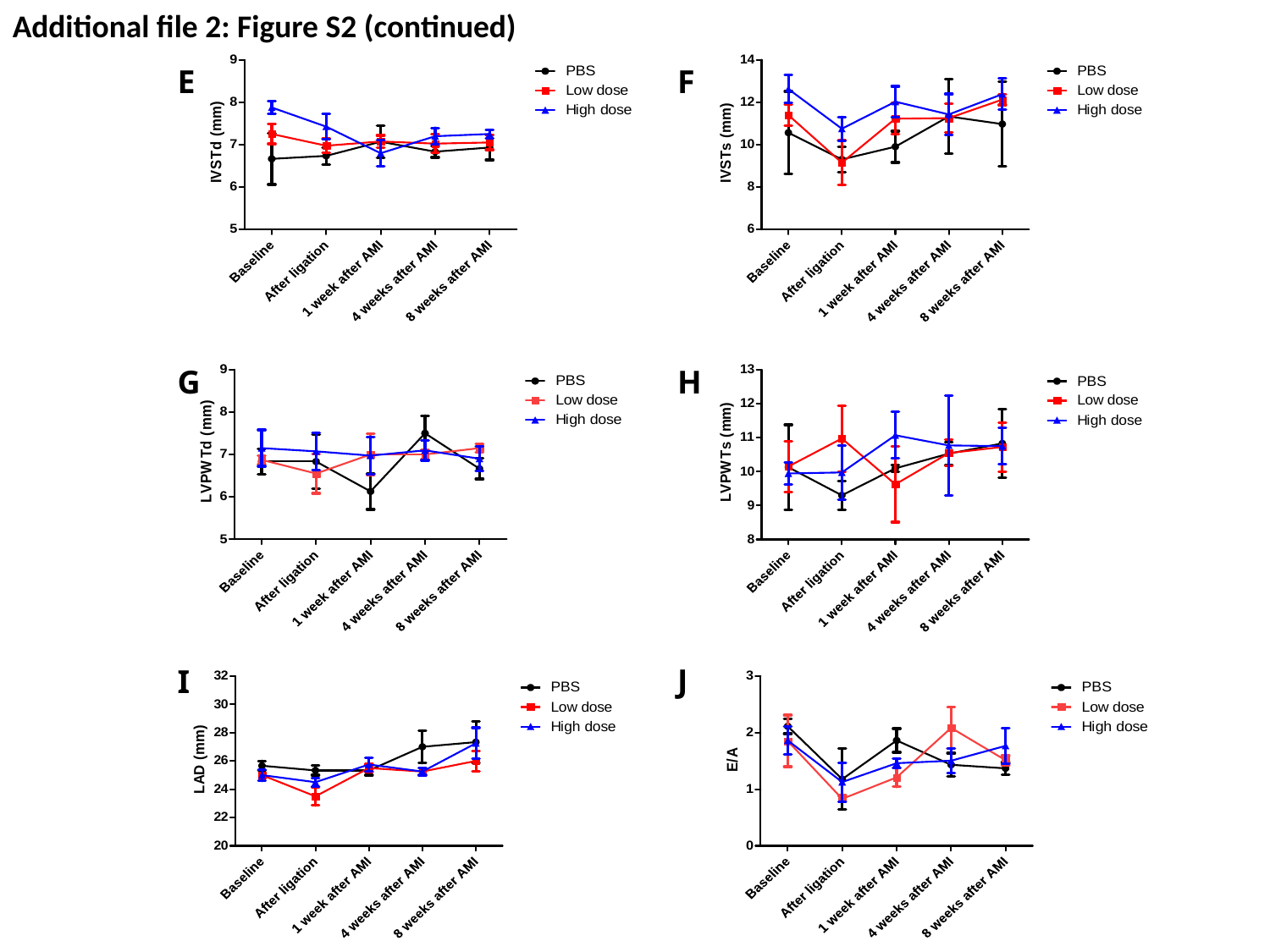

Additional file 2: Figure S2 (continued)
E
F
G
H
J
I

Supplement: Supplementary file 3 — Figure S2. Effects of intravenous injection of allogeneic UC-MSCs on cardiac structure and diastolic function after AMI. Left ventricular end-diastolic diameter (A), left ventricular end-systolic diameter (B), left ventricular end-diastolic volume (C), and left ventricular end-systolic volume (D). Intraventricular septal diastolic thickness (E), intraventricular septal systolic thickness (F), left ventricular posterior wall diastolic thickness (G), and left ventricular posterior wall systolic thickness (H). Left atrial diameter (I). Cardiac diastolic function: E/A ratio (J). Data are presented as the mean ± SD (PBS group n = 3, low-dose group n = 4, and high-dose group n = 4). *P < 0.05 (ZIP 298 kb) [file 13287_2018_888_MOESM3_ESM.zip › Additional file 2 Figure S2 (continued).pptx]

## Slide 1
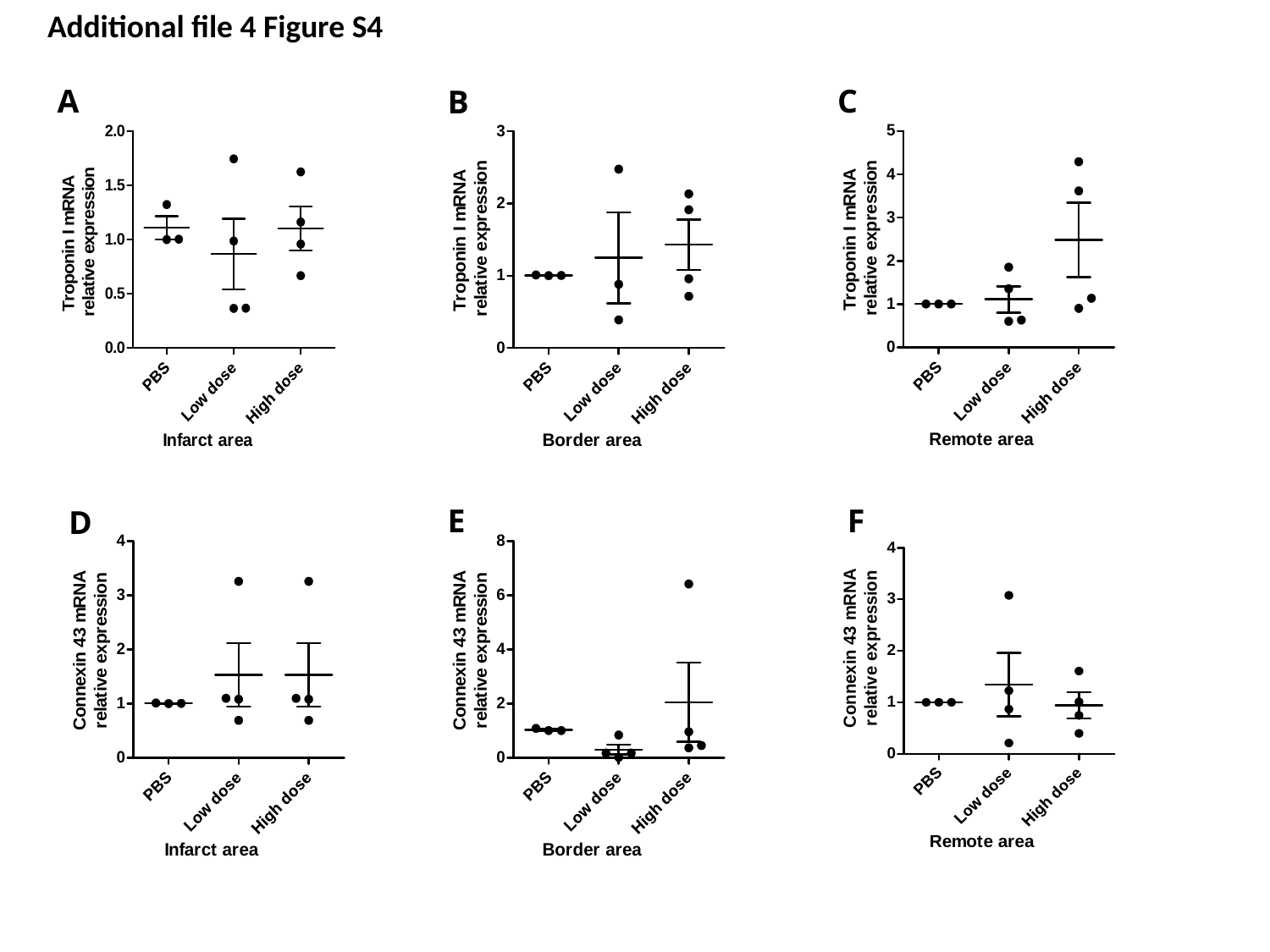

Additional file 4 Figure S4
A
C
B
E
F
D

Supplement: Supplementary file 5 — Figure S4. Intravenous injection of allogeneic UC-MSCs did not impact mRNA expression levels of the cardiac function biomarkers troponin I and connexin 43 in the infarct area (A,D), border area (B,E), or remote area (C,F) of LV myocardium at week 8 after AMI. (PPTX 157 kb) [file 13287_2018_888_MOESM5_ESM.pptx]

## Slide 1
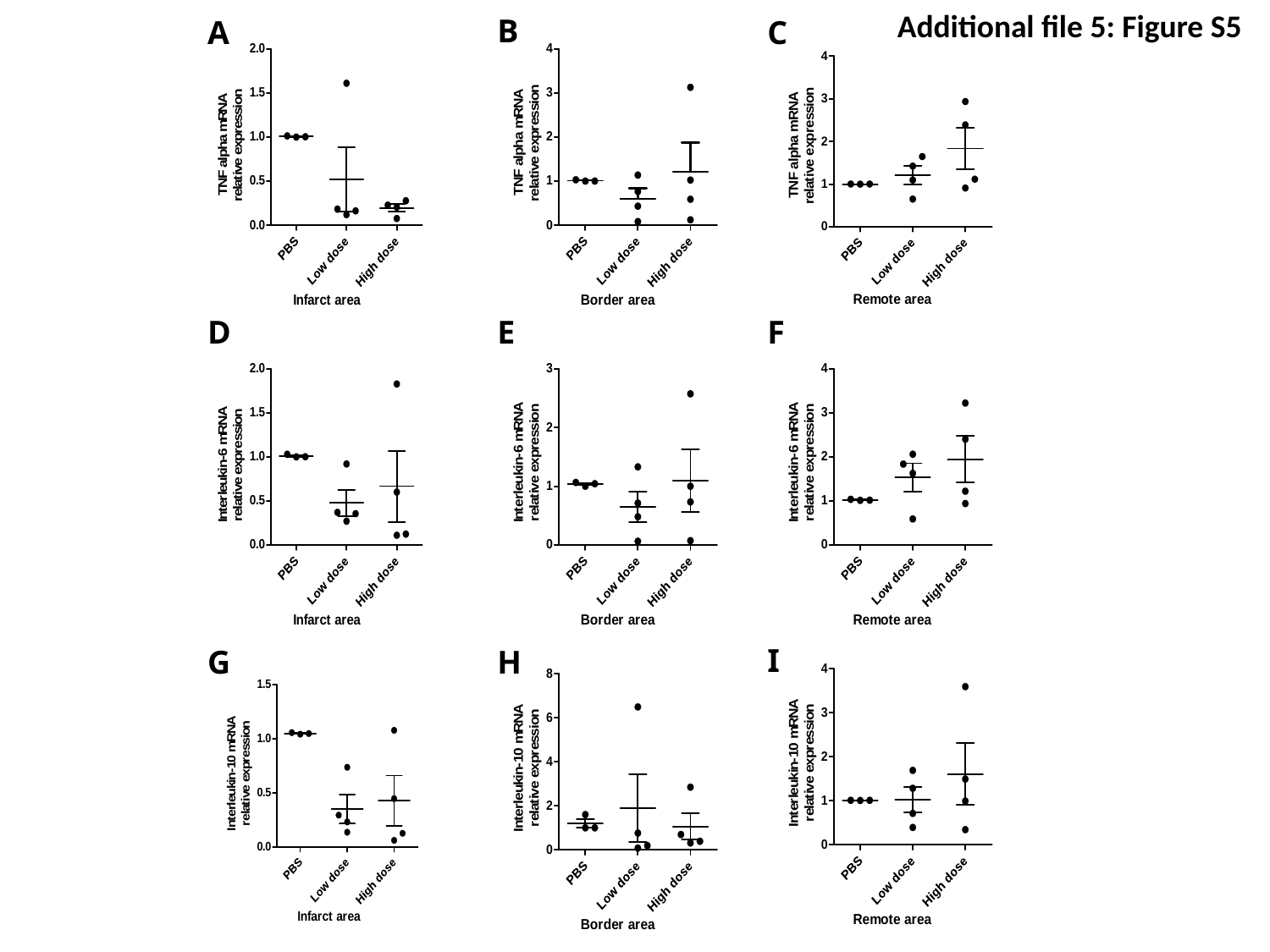

Additional file 5: Figure S5
B
A
C
D
E
F
I
G
H

Supplement: Supplementary file 6 — Figure S5. Intravenous injection of allogeneic UC-MSCs did not affect mRNA expression level of the inflammation factors TNF alpha, IL-6, IL-10, and TGF-beta in the infarct area (A,D,G,J), border area (B,E,H,K), or remote area (C,F,I,L) of LV myocardium at week 8 after AMI. (ZIP 248 kb) [file 13287_2018_888_MOESM6_ESM.zip › Additional file 5 Figure S5.pptx]

## Slide 1
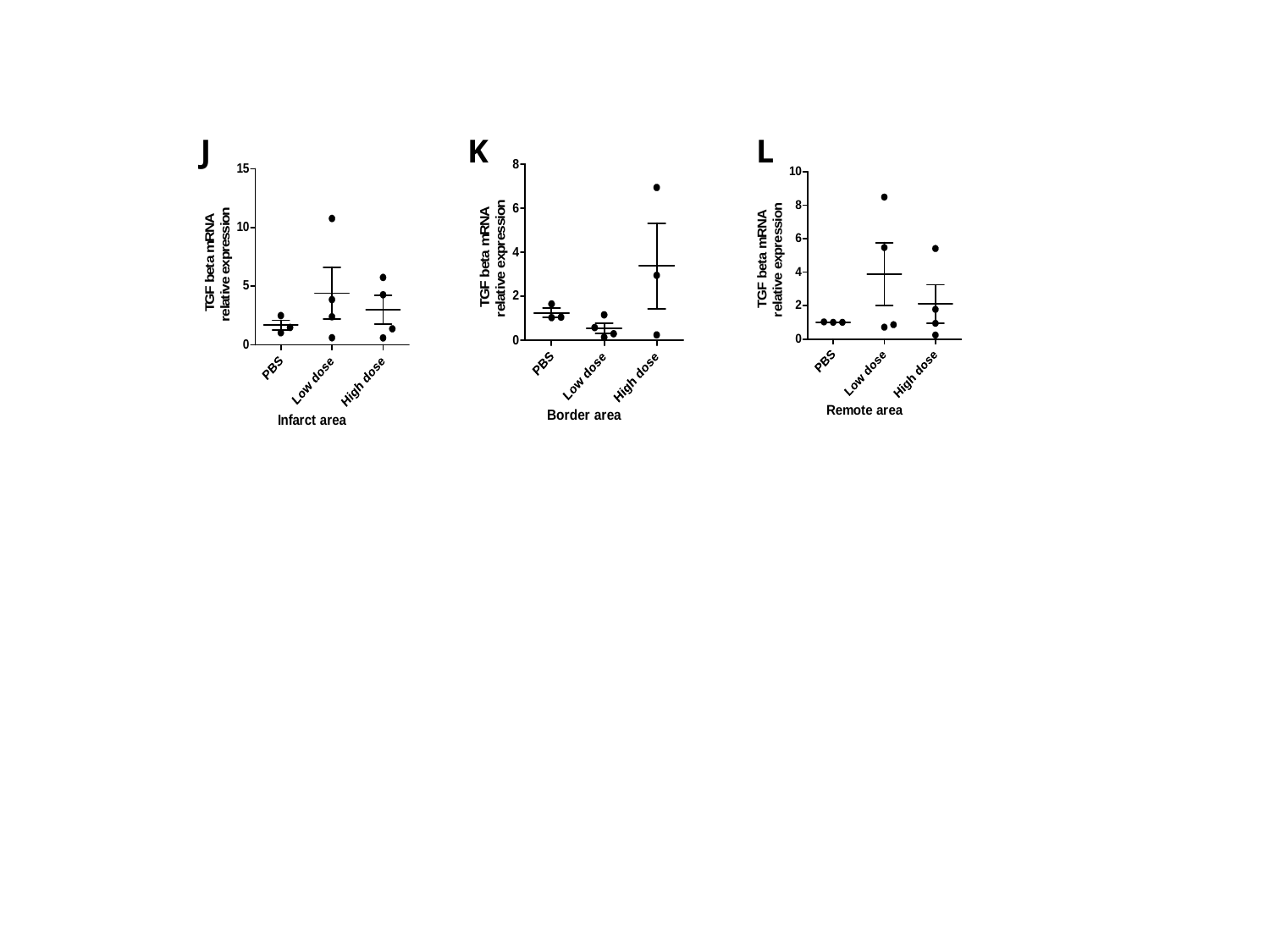

J
K
L

Supplement: Supplementary file 6 — Figure S5. Intravenous injection of allogeneic UC-MSCs did not affect mRNA expression level of the inflammation factors TNF alpha, IL-6, IL-10, and TGF-beta in the infarct area (A,D,G,J), border area (B,E,H,K), or remote area (C,F,I,L) of LV myocardium at week 8 after AMI. (ZIP 248 kb) [file 13287_2018_888_MOESM6_ESM.zip › Additional file 5 Figure S5 (continued).pptx]

## Slide 1
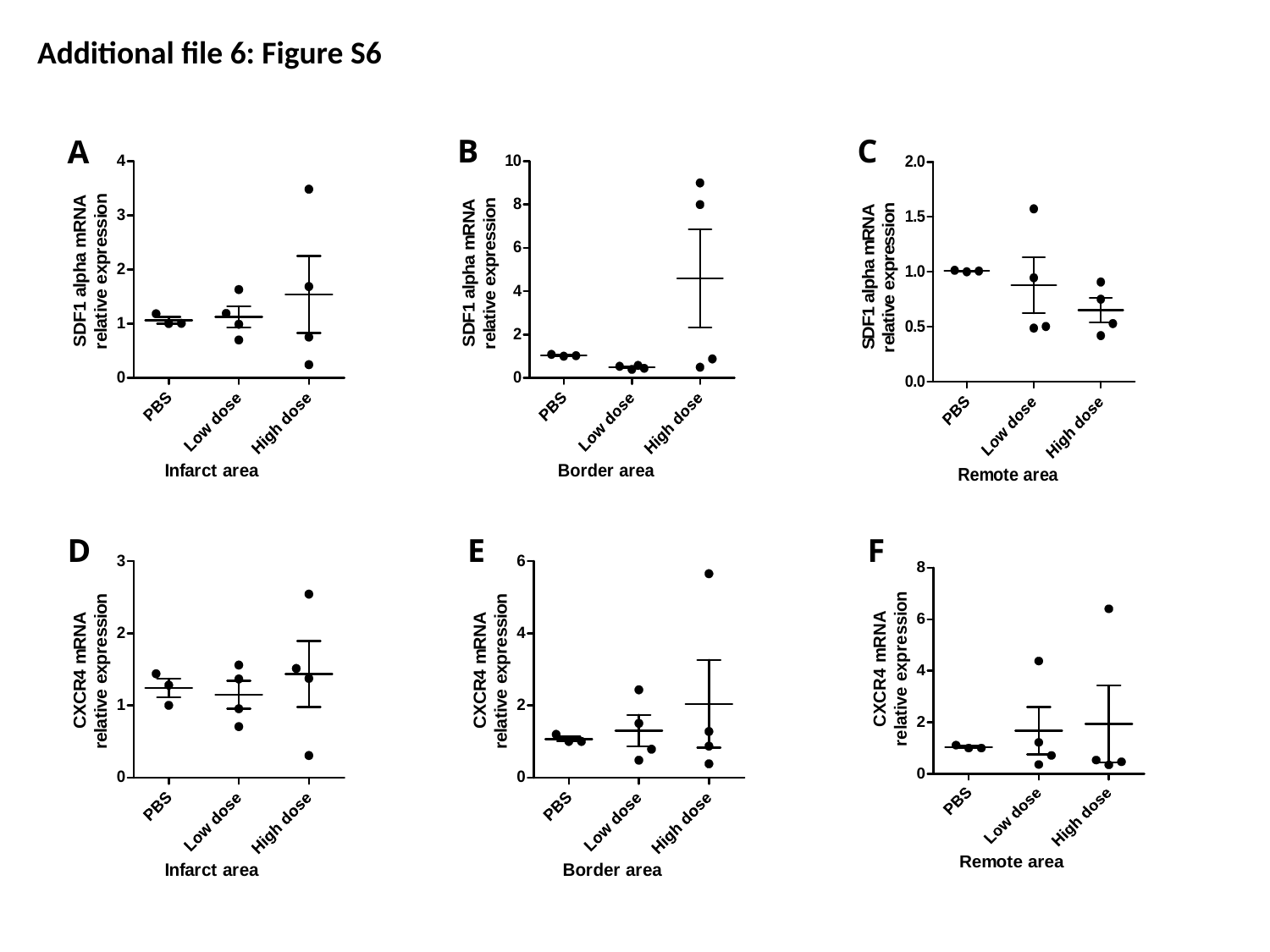

Additional file 6: Figure S6
B
C
A
D
E
F

Supplement: Supplementary file 7 — Figure S6. Intravenous injection of allogeneic UC-MSCs did not affect mRNA expression of the chemotaxis factors SDF1-alpha and its receptor CXCR4 in the infarct area (A,D), border area (B,E), or remote area (C,F) of LV myocardium at week 8 after AMI. (PPTX 151 kb) [file 13287_2018_888_MOESM7_ESM.pptx]

## Slide 1
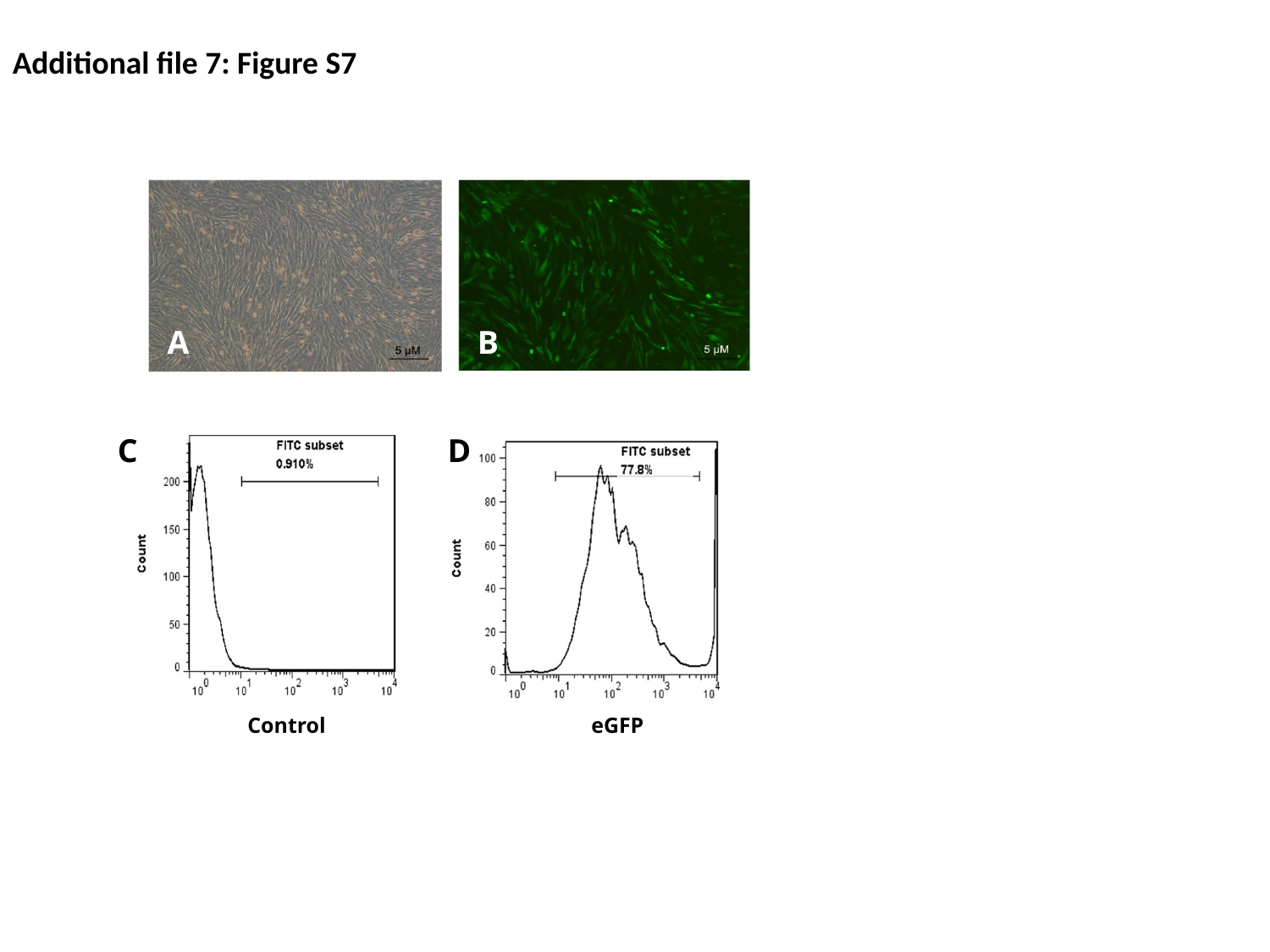

Additional file 7: Figure S7
A
B
C
D
Control
eGFP

Supplement: Supplementary file 8 — Figure S7. Transfer of lentivirus-eGFP into UC-MSCs. Representative phase-contrast (A) and fluorescence (B) microscopic photograph of UC-MSCs transduced with lentivirus-eGFP. (C,D) Flow cytometric analysis of the transfection efficiency of lentivirus-eGFP into UC-MSCs. Scale bar = 5 μm. Data are representative of three independent experiments. (PPTX 795 kb) [file 13287_2018_888_MOESM8_ESM.pptx]

## Slide 1
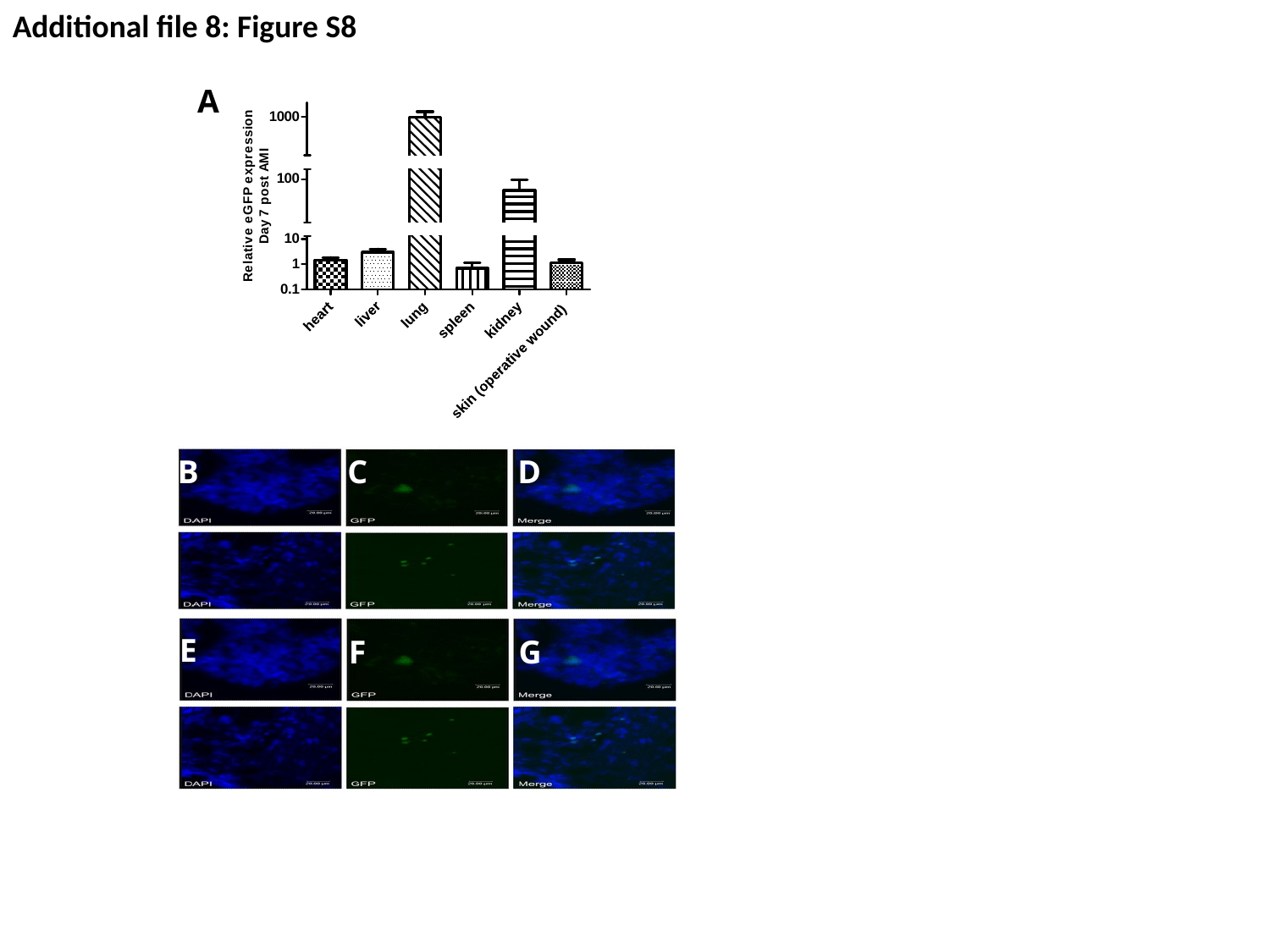

Additional file 8: Figure S8
A
B
C
D
E
F
G

Supplement: Supplementary file 9 — Figure S8. In vivo trafficking of intravenous injection of allogeneic UC-MSCs-eGFP 7 days after AMI induction. (A) Biodistribution pattern of UC-MSCs-eGFP measured by DNA relative expression of eGFP in many tissues (heart, lung, liver, spleen, kidney, wound operation of skin). (B–D) Immunofluorescence on eGFP (green) and nuclei (blue) in lung tissue. (E–G) Immunofluorescence on eGFP (green) and nuclei (blue) in peri-infarct heart tissue. Scale bar = 20 μm. (PPTX 783 kb) [file 13287_2018_888_MOESM9_ESM.pptx]

## Slide 1
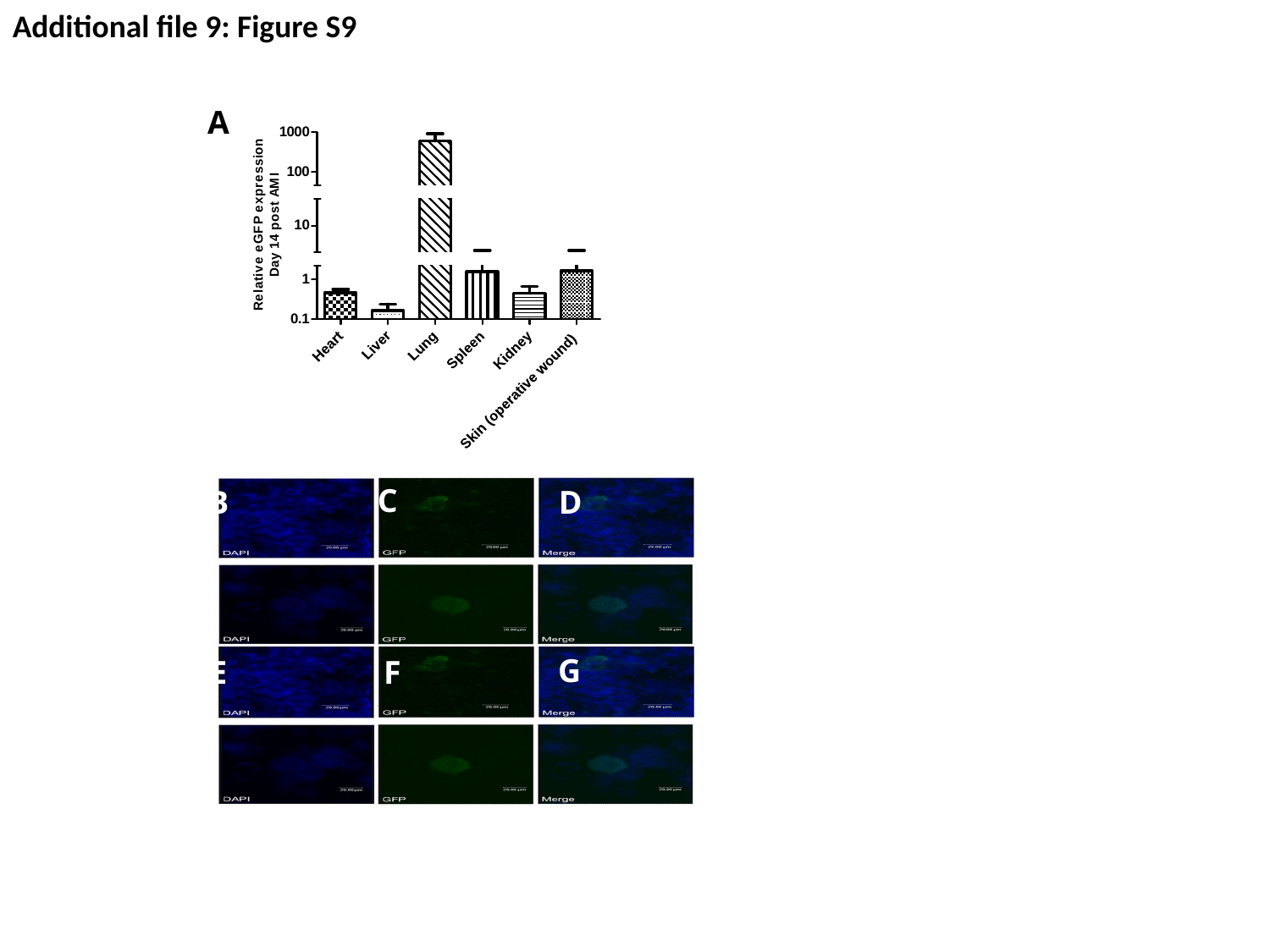

Additional file 9: Figure S9
A
C
B
D
G
E
F

Supplement: Supplementary file 10 — Figure S9. In vivo trafficking of intravenous injection of allogeneic UC-MSCs-eGFP 14 days after AMI induction. (A) Biodistribution pattern of UC-MSCs-eGFP measured by DNA relative expression of eGFP in many tissues (heart, lung, liver, spleen, kidney, wound operation of skin). (B–D) Immunofluorescence on eGFP (green) and nuclei (blue) in lung tissue. (E–G) Immunofluorescence on eGFP (green) and nuclei (blue) in kidney tissue. Scale bar = 20 μm. (PPTX 1467 kb) [file 13287_2018_888_MOESM10_ESM.pptx]
