# Supplementary material for: Intravenous injection of allogeneic umbilical cord-derived multipotent mesenchymal stromal cells reduces the infarct area and ameliorates cardiac function in a porcine model of acute myocardial infarction
Source: Stem Cell Res Ther. 2018 May 11;9:129. doi: 10.1186/s13287-018-0888-z (PMC5948807; doi:10.1186/s13287-018-0888-z)
Supplement: Supplementary file 4 — Figure S3. Histological images of left ventricular basal, middle, and apex cross-sections after TTC staining in the three groups PBS (n = 3), low-dose group (n = 4), and high-dose group (n = 4) at 8 weeks follow-up. Schematic infarct distribution shows that the infarction (white color) is located in the anterior and anteroseptal segments of the heart. (PPTX 6390 kb) [file 13287_2018_888_MOESM4_ESM.pptx]

## Slide 1
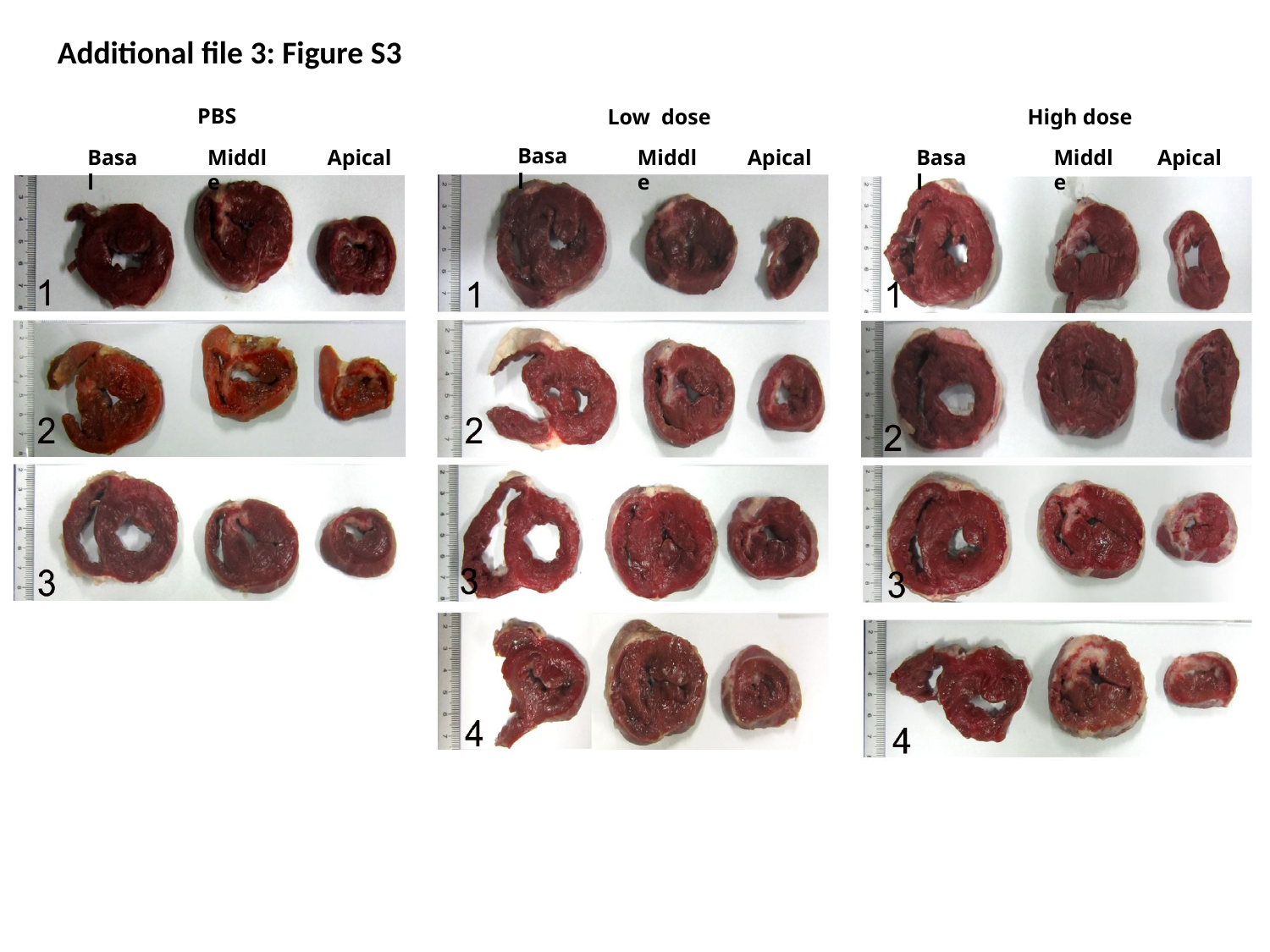

Additional file 3: Figure S3
PBS
Low dose
High dose
Basal
Basal
Middle
Apical
Middle
Apical
Basal
Middle
Apical
